# Word embeddings as autonomous predictors in materials design—the effect of inherent variability on information transfer

**DOI:** 10.1186/s13321-025-01149-3

**Published:** 2026-02-09

**Authors:** Jana Radaković, Katarina Batalović, Nikola Novaković

**Affiliations:** Department of Nuclear and Plasma Physics, “VINČA” Institute of Nuclear Sciences - National Institute of Republic of Serbia, University of Belgrade, Mike Petrovića Alasa 12-14, POB 522, 11001 Belgrade, Serbia

**Keywords:** In-silico materials design, NLP-based autonomous predictors, Word2vec, Digital materials, Word embeddings in materials design, Materials stability, Formation energy predictions, Random forest regression

## Abstract

We propose that word embeddings of atoms derived from scientific literature are revisited as autonomous machine learning predictors in materials design. If static word embeddings encode comprehensive physicochemical information, joined embeddings of chemical elements constituting a chemical compound represent a viable source of physicochemical knowledge. Nevertheless, static word embeddings are susceptible to variability due to information heterogeneity within training material. We analysed whether variability occurs in embeddings affiliated with physicochemical entities, including explicit atoms, and whether it affects therein-encoded domain-specialized information or inhibits the information transfer. Results demonstrate the substantial variability in individual atomic embeddings, which is highly dependent on vocabulary terms selected for language modelling. Regardless, variability does not obstruct the mapping of materials' composite predictors into physicochemical properties when joined atomic embeddings are implemented within a regression model estimating the compound stability by predicting its formation energy. Moreover, the encoded information and the model's predictive performance maintained stability following compound vector calibration via dimensional reduction.

Scientific contribution

The magnitude of variability in word embeddings of physicochemical entities, including chemical elements, occurring due to information heterogeneity in complementary training material of materials science, chemistry, and physics scientific literature was observed and quantified. The research shows that notable variability of vectorial representations of chemical elements does not obstruct the underlying statistical properties, nor does it inhibit the information transfer. Accordingly, regardless of their origin, conjoined atomic embeddings representing chemical compounds facilitate stable predictive performance when implemented within a regression model.

## Introduction

Due to its potential for design and discovery of novel inorganic and organic materials, natural language models have been extensively studied and applied in research fields beyond computational linguistics, such as materials science, chemistry, or physics [1–18]. Even advancements in some of the medical diagnostic methods and the development of new biological materials have been relying on recently developed large language models (LLM) and established natural language processing (NLP) techniques [19–29].

Providing a language model with a large amount of highly specialized materials science, chemistry, or physics-related documents facilitates the generation of high-dimensional vectorized representations – word embeddings – of frequently occurring domain-specific terms, including atoms, molecules, inorganic and organic compounds, formulae, experimental methods, and alike. It is claimed that the word embeddings of such physicochemical entities inherently encode extensive and diverse range of physicochemical concepts, general chemical knowledge, and materials’ structural complexity, offering a compact mathematical format that can serve as an indiscriminate, task-agnostic input for machine learning (ML) models focused on solving materials science specific objectives.

The seminal work on the subject, performed by Tshitoyan et al. [8], demonstrated how word embeddings of chemical elements could serve as feature vectors in quantitative machine learning models and can be used to analyze the potential of materials thermoelectric properties and to predict their overall stability by computing their respective formation energy. Their research highlights that if the chemical information is sourced from word embeddings of the constituent chemical elements of a chemical compound, the resulting composite compound embedding effectively becomes a computationally derived composition-based feature vector encompassing the properties of its constituent atoms. Unlike conventional feature vectors, which are manually designed descriptors offering discrete solutions to distinctive research assignments, these high-dimensional, fixed-length word embeddings of chemical elements are inherently task-agnostic and, accordingly, adaptable tools for addressing a range of diverse problems in materials design.

If text-based embeddings are to serve as autonomous feature vectors in machine learning applications oriented towards solving chemistry or materials science-related challenges, the embeddings have to be of exceptional quality. The reason for this lies in their direct applicability in automated language-based materials discovery pipelines and high-throughput screening processes, where millions of hypothetical compounds are simultaneously being discovered, tested, and screened for either one or multiple different applications. Due to the complexity of such workflows, which generally lack direct expert supervision or intervention, they require highly reliable input to produce accurate, precise, and reproducible outcomes. Conversely, even slight variations could compound into significant differences in predicted physicochemical properties, leading to the favorable candidates being incorrectly filtered out or, more seriously, to flawed candidates advancing to unnecessary experimental validation. Besides, when embeddings are predisposed for further fine-tuning aimed at specialized tasks, inconsistent base representations of chemical elements and compounds could prevent models from converging or would require extensive retraining. Failing to meet the high standards required by automated materials design, inconsistent representations of chemical elements and compounds can yield contradictory conclusions about the same material across different workflows. This raises several fundamental questions: what exactly defines the quality and effectiveness of word embeddings in this context, and how can they be quantified in terms of materials design? To provide an answer, it would be necessary first to register the likely complexities occurring when using word embeddings as a specialized set of features. Key factors that directly dictate the performance of a machine learning model trained on such features, and the predictions for which the embeddings could be tasked, include the very language model by which the word embeddings are generated, the dimensionality of the embedding vectors, the nature of them being static or context-dependent, and other configurable parameters, majority of which are easily adjustable during the initial stages of a computational experiment.

Beyond these adjustable factors, examining the potential sources of inconsistency within the word embeddings themselves is also critical. As evidenced by prior research, the quality of the information provided in training literature, the characteristics of the training corpus, as well as its size play a major role in determining the functionality of the word embeddings. [30–33] Several studies, however, illustrate how, irrespective of the quality of training procedure or other tunable factors, otherwise static word embeddings could become highly mutable under certain conditions. In essence, high-dimensional vector representations of words, which were expected to remain consistent across various types of comparable training vocabularies, have been shown to display variable properties. Accordingly, contingent on the level of variability, the behavior of word embeddings within machine learning tasks and their downstream objectives could be considered unreliable and, in the context of highly specialized domains, where precision and reproducibility are paramount, inapplicable [34–39].

One of the initial definitions of the variability of word embeddings was presented in the work of Burdick et al.:[35] “stability^1^ is defined as the percent overlap between nearest neighbors in different embedding spaces”. In other words, the variability reflects the inconsistency in a set of most similar tokens of one token randomly selected from vocabularies generated using different training documents. Examples of this phenomenon have been documented across various documents and diverse languages. Antoniak and Mimno[34] found that embedding algorithms Word2vec[31], GloVe[40], PPMI[32], and LSA[41], are all sensitive to the number of documents used to train the NLP model, the context within those documents, the presence or absence of specific documents, and the corresponding size of the training corpus. They emphasized that lengthier documents increase the variability of word embeddings. Wendlandt et al.[38] compared the variability of embeddings generated using Word2vec, GloVe, and PPMI algorithms across different domains of news-containing documents. They also included the frequency of words, as it changes with respect to the studied domain and is reflected in the representation of words. The authors found that the GloVe algorithm produces the most stable embeddings with respect to the frequency when observed in a single domain. PPMI achieved similar stability, while Word2vec proved to be highly dependent on both the frequency and the selected domain, with the medium-frequency words exhibiting surprisingly marked variability. On the contrary, Pierrejean and Tanguy [37] observed the highest variability among the nearest neighbors of the words with the lowest or highest frequency. Besides the obvious factors affecting the downstream variability of word embeddings, another practical aspect was reported by Leszczynski et al.[36] They demonstrated the effect of the number of embedding dimensions, which implied that depending on the training algorithm and corpus type, the variability should plateau at approximately 100 dimensions.

Briefly outlined studies address the variability of word embeddings derived from non-specialized training literature. While the results, though obtained via rather obsolete techniques, are versatile, they collectively underscore the need for exceptional attentiveness to the selection strategy of training documents and their subsequent effect. The more modern, natural language-based techniques applied in materials science, chemistry, and physics also accentuate the corpus-centered approach in generating outstanding text representations of chemical entities, irrespective of varying methodologies used for ML training. The prevailing rationale is that for natural language embeddings, and by extension word embeddings, to effectively encode the information conveyed by the training corpus, the information must be pervasive and well-represented in the literature. To achieve the excellence, depending on the research goal, the training corpora is either indiscriminately comprised of a very broad range of general documents or narrowly focused on one subject matter. Furthermore, much of the existing research [4, 10, 15, 19, 42–44], including the limited studies that utilize word embeddings as feature vectors [21, 25, 27, 28, 45], perceive the investigated information of interest as indisputably encoded within the vectorized representations of domain-specific terms, regardless of their origin. Consequently, these studies often highlight comparison of benchmark properties predictions obtained using different language embeddings – such as Word2vec, MatSciBERT[4], MatBERT[15], and others – while disregarding the fact that they have been trained using documents conveying potentially dissimilar information, or those originating from different domains and corpora of various lengths.

Nevertheless, the research on applications of language models in these highly specialized domains is still in its infancy, with current efforts primarily focused on optimizing and fine-tuning language models for similarity-based, task-specific objectives, such as named entity recognition or recognition and extraction of candidate materials for targeted applications [10]. The effects of distinct or complementary scientific literature, used as training documents, on representations of chemical entities, therefore, remains underexplored. In particular, the magnitude of potential variability originating from a variety of training corpora in materials science and chemistry-related word embeddings, including those representing explicit inorganic and organic compounds, and its downstream effects on the predictive ML tasks has yet to be reported.

If the heterogeneity of training documents is the primary source of variability in word embeddings, a certain degree is undoubtedly expected to occur in materials science and chemistry-related word embeddings when trained using multifaceted and general knowledge. If the training material is sufficiently multifarious, learned representations should expectedly fluctuate; however, we reasoned that if documents are sourced from a thematically similar milieu of analogous or complementary knowledge, the previous conclusion is not a straightforward one. Guided by this reasoning, throughout the first portion of this manuscript, we focused on investigating the potential for variability in materials science-affiliated word embeddings trained in three distinct training environments generated using publicly available research manuscripts from the domains of chemistry, physics, and materials science. The degree of estimated variability further served as an estimator of the effects related scientific data, as distinct training environments, had on the ML model’s capacity to capture and exploit the domain-specialized technical and non-technical details.

The second part of the manuscript focused on the potential of selected language model for the conservation of information compiled from various domain documents, the accessibility of information via a machine learning model, the tractability of high-dimensional information carriers by rudimentary manipulation, and the information transfer from a complex numerical format into an intelligible real-world physicochemical value. We analyzed the extent to which word embeddings of chemical elements encapsulate meaningful information and whether they accurately reflect the multifaceted nature of these elements. To achieve this, following the methodology outlined by Tshitoyan et al. [8], we utilized the joined word embeddings of chemical elements constituting a chemical compound as a source of highly specialized chemical knowledge. In this framework, composite compound embeddings served as composition-based feature vectors reflective of information intrinsic to the text representation of their constituent elements, which, according to research, are susceptible to significant variations. This experimental design enabled us to assess whether ensuing variations in word embeddings of atoms have a measurable effect on the predictive performance of machine learning models estimating the stability of compounds if the respective composite embeddings are employed as feature vectors. In addition, we tested whether these high-dimensional composite embeddings preserve the rooted information after being subjected to feature vector calibration. This was performed by eliminating all features that do not correlate with the selected response variable, which compressed the training matrix to an exclusive set of information-rich predictors.

The results of this investigation provide deeper insights into the reliability and generalizability of text-derived representations in computational chemistry and materials science. To once again highlight the importance of such actions, in large-scale automated materials design workflows, where machine learning models make decisions with minimal external supervision, understanding the source of variability and controlling its outcomes becomes a critical issue. When distinct, potentially interchangeable materials are tested for future application based on the similarity of their text-derived representations, the variability of their representation within the context of the definition provided by Burdick et al.[35] is an attribute that needs to be assessed. Representations of materials that perform well on benchmarks but exhibit high variability may lead to extreme physicochemical inconsistencies or even fail when applied in novel chemical spaces or under any shifting input conditions. This particular issue can critically affect autonomous laboratories, where, in addition to exceptional machine learning predictions, the synthesis of materials and their subjugation to costly experiments also rely on the quality of input data. By identifying the sources of the variability of atomic and compound word embeddings, one could develop methods to control its consequences and increase the functionality of word embedding representations of atoms, molecules, and organic and inorganic compounds in in-silico materials design. Such advancements would enable word embeddings to efficiently serve as robust feature vectors, truly encapsulating the information necessary for deriving new insights and data.

## Results

### Defining variability of word embeddings and the concept of embedding subspace

To investigate the phenomenon of variability in materials science and chemistry-affiliated word embeddings, the three distinct corpora were generated, each representing a dominant discipline in experimental and in-silico materials design – chemistry, physics, materials science – alongside a fourth corpus comprising a mixture of these fields. The compiled manuscripts were considered sufficiently thematically diverse while providing complementary physicochemical information that could be encoded in word embeddings of chemical formulae, words, and phrases. This framework enabled the analysis of the extent of vectorial divergence among word embeddings of high-frequency expressions commonly reported within each research discipline.

**Fig. 1:**
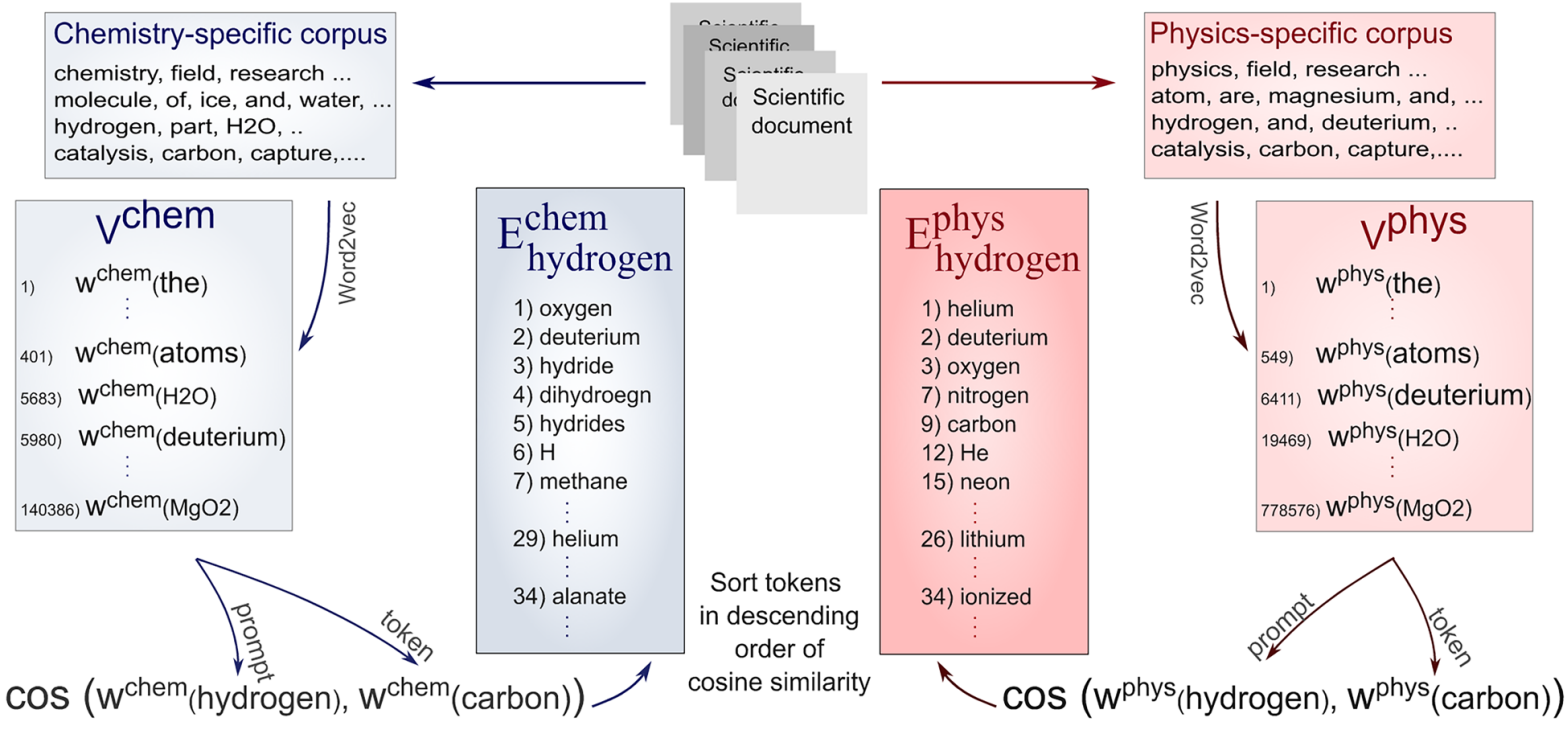
Generation schema of two hydrogen embedding subspaces obtained using chemistry-specific and physics-specific vocabularies ${\mathrm{E}}_{\mathrm{p}}^{\mathrm{d}}$: ∀ t, sort[cos sim(w^d^(p), w^d^(t|p))] ↓

To reiterate, the observed variability reflects inconsistencies among the sets of nearest neighbors of one token when randomly drawn from different vocabularies generated using different training documents [34, 35, 38, 46]. A set of nearest neighbors of a token is defined as a finite collection of tokens assembled from the same vocabulary, which exhibit similarity to the initial token based on some predefined metrics. For a meaningful comparison between two sets, they should have comparable dimensions and be drawn from a collection of tokens with a similar distribution. These preliminary requirements, whose effects are explored in detail, motivated the introduction of the concept named embedding subspace, the generating process of which is schematically illustrated in Fig. 1.

Consider two independent domain-specific vocabularies (V^d^): a chemistry-specific (V^chem^) and a physics-specific vocabulary (V^phys^). Both vocabularies consist of word embeddings of terms characteristic of their respective research disciplines (e.g., atom, catalysis, superconductivity, H2O, hydrogen, He, etc.), all with varying average occurrence frequency across domains. By selecting a term with a relatively high occurrence frequency – referred to as a prompt – one can extract its word embedding from both domain vocabularies (w^d^(p)), following which the word embeddings of tokens most similar to it could be identified within both domains using cosine similarity measure w^d^(t|p): cos sim (w^d^(p), w^d^(t)). This measure enables ranking extracted tokens relative to their proximity to the prompt, thereby generating a unique, sorted embedding subspace for selected prompts in both domains (${\mathrm{E}}_{\mathrm{p}}^{\mathrm{d}}$). The divergence between two domain-specific embedding subspaces is quantified by computing the Szymkiewicz-Simpson overlap coefficient, which captures the degree of observed variability of included word embeddings. As will be demonstrated, this variability is significantly influenced by the structural composition of the embedding subspaces and the origin of the parent domain of training documents.

#### The intersection of domain-specific vocabularies

**Fig. 2:**
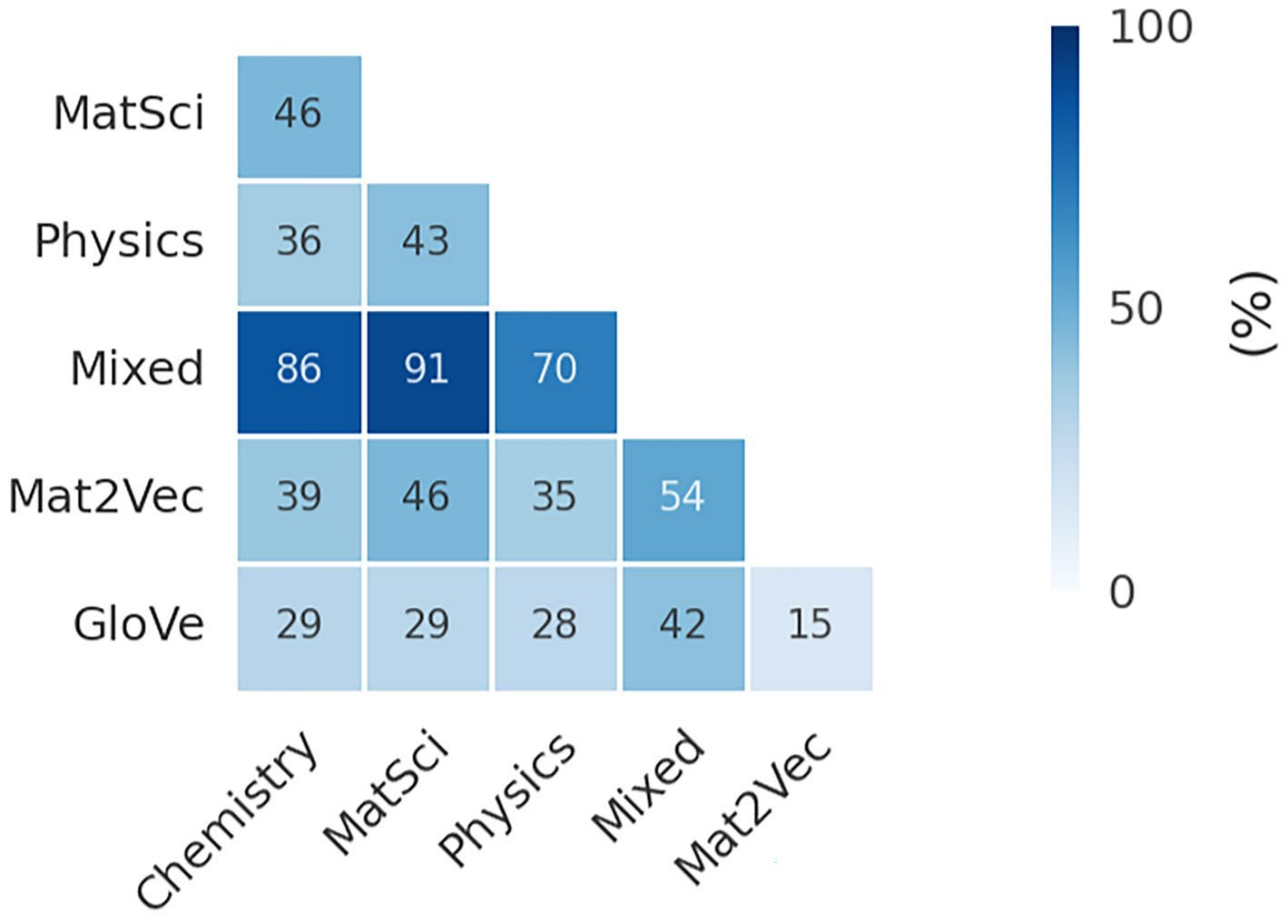
Szymkiewicz-Simpson overlap coefficients of unrestricted vocabularies

Before proceeding to analyze the intersection of thus generated embedding subspaces, we will briefly examine the intersection of initially obtained vocabularies and provide the fundamental limitations inherent to their usage in this context. As indicated in Fig. 2, where the Szymkiewicz–Simpson overlap coefficients of custom vocabularies (Chemistry, Physics, MatSci, Mixed) alongside the baseline vocabularies (Mat2Vec and GloVe) are quantitatively presented, the degree of intersection between vocabularies manifests a remarkable heterogeneity, with only a fraction of tokens shared across domains, regardless of the content comparability and complementarity exhibited by the training corpora.

The overlap coefficients of the baseline vocabularies, GloVe and Mat2Vec, were found to be only 15%, resulting in approximately 60,000 GloVe terms intersecting the domain-specific Mat2Vec. This observation was consistent with the ontological construction of vocabularies and the fact that Mat2Vec systematically excludes terms lacking explicit function in materials science. Furthermore, approximately 30% of GloVe vocabulary was fully contained within each custom vocabulary, excluding Mixed, which also reflected the differentiation of linguistic composition among the source training documents. Considering that the custom vocabularies encompass nearly twice the terminological density of Mat2Vec (as indicated in Table 3 of the Methods section), this 30% proportion of GloVe terms is considered an invariant parameter relative to all comparative vocabularies.

A domain-specific vocabulary, Mat2Vec, was expected to correlate highly with other custom vocabularies, particularly those originating from the materials science domain. Contrary to expectations, the intersection coefficients between Mat2Vec and any custom vocabulary were observed to plateau at about 50%. Equally unexpected was the finding that lexical overlap between the MatSci and Physics, the MatSci and Chemistry, and between the Physics and Chemistry also did not exceed 50%. This phenomenon was attributed to the heterogeneous distribution of frequently occurring physicochemical terms, such as chemical formulae and units of measurement, appearing in manuscripts of studied domains. The sole exception was noted in the Mixed vocabulary, which was self-explanatory, given the composite structure of the training corpus, as well as its size, which effectively compensates for the lexical diversity. A further striking observation was that the exclusion of all terms except chemical formulae from vocabularies gave rise to pruned vocabularies with a markedly reduced number of tokens and an even more reduced overlap percentage.

The overlap of vocabularies, as quantified, analyzed, and here presented, is a moderately informative metric. Notably, it fails to derive any insightful information about the underlying structural composition of vocabularies, the configuration of intrinsic word embeddings, or their potential efficacy in diverse operational environments. Consequently, the scope of variability analysis had to be expanded to include the interpretation of its impact on these embeddings in a context where representative physicochemical entities are present in great numbers.

#### The intersection of embedding subspaces

The structural composition of vocabularies and the variability of contained word embeddings were analyzed from the perspective of embedding subspaces generated using prompts frequently documented in materials science, chemistry, and physics research, as cataloged in Table 1. Complementary embedding subspaces were also constructed using the first 95 chemical elements from the periodic table (spanning from hydrogen to americium), providing that their respective embeddings served as feature vectors subsequently integrated into a regression model designed to predict the stability of chemical compounds, elaborated in later sections of this manuscript. This analytical approach revealed both qualitative and quantitative differences among distributions of nearest neighbors of selected prompts, which manifested as significant variations in the computed overlap percentages.

**Table 1:** High-frequency prompts used for embedding subspaces generation

Band gap, catalyst, anode, cathode, batteries, enthalpy, membrane, hydride, supercapacitors, dielectric, insulin, glucose, acetylation, ethyne, hydrocarbon, hydrogen storage, photocatalyst, ozone, ferroelectric, ferromagnetic, antiferromagnetic, perovskite, thermoelectric, lithium ion, intermetallic, photovoltaic, superconducting, FCC, BCC, metallocene, laves phases, zintl phases, ceramics, polymerization, chalcogenide, noble gas, actinide, ion batteries, metallocene, insulator
LiCoO_2_, MgH_2_, Al2O_3_, TiO_2_, BiFeO_3_, Bi2Te_3_, ZnO, SiO_2_, PbTe, H_2_O, H_2_O_2_, AlH_3_, C_6_H_12_O_6_, C_2_H_2_, ZrNi, SnSe, NiFe, IrMn, ZrO_2_, Cr_2_O_3_
Hydrogen, helium, lithium, berylium, boron, carbon, nitrogen, oxygen, fluor, …, americium

**Fig. 3:**
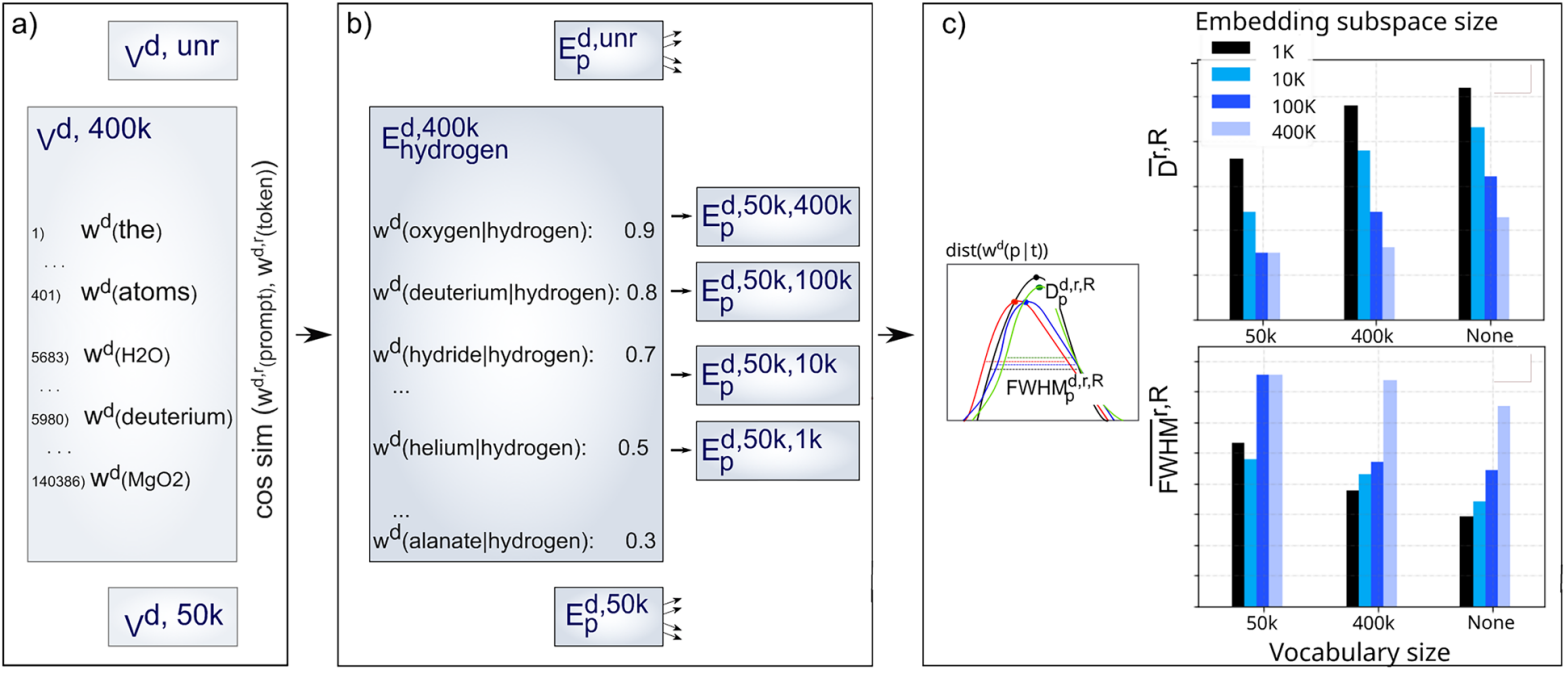
**a** Vocabularies size restrictions; **b** Embeddings subspaces restrictions; (c) Average maximal similarity scores and average full width at half maximum calculated for all imposed restrictions

Each constructed vocabulary contained between about 500,000 and 900,000 tokens representing various lexical categories (for more details on the corpora and vocabulary structure refer to the Methods section). While certain tokens exhibited high domain specificity, reflecting the semantic nuances of their originating field, others demonstrated minimal domain relevance. To mitigate the influence of this lexical heterogeneity and to simultaneously eliminate the effects of vocabularies’ variable length on the intersection of embedding subspaces, the number of participating tokens from each vocabulary was controlled by setting the three distinct vocabulary configurations (V^d, r^; d: domain, r = (unr, 400k, 50k)), as visualized in Fig. 3(a). In the baseline configuration (V^d, unr^), the vocabulary from which nearest neighbors of high-frequency prompts were sampled to populate the corresponding embedding subspaces was kept unrestricted. Each token had an equal probability of being identified as the nearest neighbor to any given prompt, regardless of its significance to the observed scientific domain. In the alternative configurations (V^d, 50k^ and V^d, 400k^), the restriction was instituted by fixing each vocabulary to the descending order of tokens’ frequency of occurrence, following which tokens’ participation was constrained to either the top 50,000 or 400,000, with all remaining lexical units discarded from the neighbor-sampling process. The selection of the 400,000 token threshold was strategically equalized with the number of tokens contained in the smallest GloVe vocabulary, facilitating the even inclusion of chemical entities with very low frequency of occurrence, yet potentially very high relevance to the domain of interest. Conversely, the 50,000 token threshold was established as a functional minimum that included words and phrases of sufficient frequency and semantic significance to contribute meaningfully to any generic downstream task. Finally, three configurations of vocabularies (V^d, unr^, V^d, 50k^, and V^d, 400k^) from which the nearest neighbors of prompts could be systematically sampled to generate the embedding subspaces respective of every studied domain were derived.

Furthermore, the restrictions were also imposed upon the size of embedding subspaces by constricting the amount of tokens to 1k, 10k, 100k, and 400k within each, thereby generating a total of twelve distinct embedding subspaces per prompt and domain (${E}_{p}^{d,r,R}$, p: prompt, R = (1k, 10k, 100k, and 400k)), as illustrated in Fig. 3(b). Such calibration of the token threshold enabled precise control over the predominant characteristics of the embedding subspaces under investigation. However, the question of whether the nearest neighbors of prompts exhibited equivalent relevance across their respective fields was beyond the analytical scope of the manuscript.

For the purpose of the comparative analysis of these embedding subspaces, distributions of cosine similarity scores, calculated between each prompt (p) and their corresponding nearest neighbor tokens (t), were derived, dist(w^d^(p|t)). From the obtained distributions, subjected to a range of vocabulary and subspace restrictions, the maximum density points (${D}_{p}^{d,r,R}$) and the full width at half maximum (${FWHM}_{p}^{d,r,R}$) of each distribution were computed. Further averaging of these values across domains and prompts (${\overline{D}}^{r,R}$ and${\overline{FWHM}}^{r,R}$), thus, provided us with critical insights into the aggregation patterns of similarities among nearest neighbors under specified conditional restrictions, as shown in Fig. 3(c). The significance of this methodological approach lied in its capacity to provide a more nuanced interpretation of the intersection of embedding subspaces. Specifically, a broad FWHM corresponded to a more extensive range of cosine similarity scores among nearest neighbors relative to their corresponding prompts, indicating a more diverse lexical collection of tokens and consequent higher probability of identifying a set of matching tokens occupying various embeddings subspaces. An increase in the FWHM, however, was accompanied by the corresponding decrease in ${\overline{D}}^{r,R}$, revealing the accumulation of tokens with lower likeness or equivalence to analyzed prompts. In contrast, a narrow peak characterized by thin FWHM correlates with an increase in${\overline{D}}^{r,R}$, signifying a higher concentration of tokens with greater similarity, I.e., those more semantically relevant to the prompt under examination.

#### The qualitative and quantitative analysis

Before proceeding to analyze the embedding subspaces, it is instructive to briefly illustrate the qualitative differences between the embedding subspaces generated using unrestricted vocabularies and those derived from vocabularies constrained to the top 1000 most frequent tokens. For that purpose, a small set of the first several tokens from embedding subspaces of an exemplary carbon prompt is presented in Table 2. A cursory inspection of the provided results revealed a moderate level of overlap, with chemical entities sharing similar physicochemical properties with carbon prompt occurring in both columns. The custom embedding subspaces predominantly featured terms signifying properties of chemical elements that exhibit high reactivity with carbon, atoms positioned near carbon in the periodic table of elements, and entities characterized by high carbon content. In contrast to the unrestricted vocabularies, each restricted vocabulary produced an embedding subspace dominated by terms explicitly related to carbon prompt as a chemical entity, reflecting the prevailing subjects of training documents. Unrestricted vocabularies, however, tended to populate embedding subspaces with terms emphasizing carbon richness, with only minimal representation of chemical elements. A notable exception was observed in the case of Mat2Vec unrestricted baseline, where, in addition to words commonly associated with carbon, the chemical formulae occurred as well. The GloVe baseline, on the other hand, reflected broader societal and natural contexts of carbon, such as carbon emissions, CO_2_, greenhouse carbon gas, carbon monoxide, and more, also underscoring the influence of training corpus composition on the resulting word embeddings and embeddings subspaces.

**Table 2:** Embedding subspaces of carbon prompt generated using unrestricted vocabularies and vocabularies restricted to first 1000 most frequent tokens

${E}_{prompt}^{domain}$	Nearest neighbors of prompts in unrestricted vocabulary	Nearest neighbors of prompts in restricted vocabulary (first 1000 most frequent tokens)
${E}_{carbon}^{matsci}$	Carbons, carbonaceous, graphitic, carbon-based, graphite, nongraphitizable, non-carbon, carbonbased, carbonaceous_material, graphitized_carbon, boron, organic_carbon, heteroatoms	Graphite, nitrogen, silicon, iron, graphene, atoms, copper, Fe, hydrogen, oxygen, Si, metals, aluminium, Ni, silica, metallic, catalyst, atom, organic, Cu, Co, steel, C, Al, Pt
${E}_{carbon}^{chemistry}$	Carbons, carbonaceous, non-carbon, nitrogen, sulfur, G-ANFs, graphitic, graphitic_carbon, organic_carbon, carbonaceous_materials, graphitic_carbon_layers, noncarbon	Nitrogen, C, hydrogen, oxygen, catalyst, N, metal, CH, organic, catalysts, iron, CO, O, H, atoms, solid, copper, electron, glucose, Fe
${E}_{carbon}^{physics}$	Nitrogen, boron, oxygen, non-carbon, carbons, graphite, sulfur, graphitic, hydrogen, magnesium, fluorine, hydrocarbon, phosphorus	Fe, CO, O, atoms, water, atomic, C, molecular, material, gas, ions, electron, atom, surface, layers, electrons, dust, formation, cm, fraction,
${E}_{carbon}^{mixed}$	Carbons, graphitic, non-carbon, carbonaceous, carbon-based, graphite, graphitization	Nitrogen, silicon, oxygen, graphene, atoms, hydrogen, Si, metal, organic, steel
${E}_{carbon}^{mat2vec}$	Carbonaceous, C11Si9, graphite, graphitic, C3Co12In4, graphitization, nitrogen,C62N, Cfe2O3, Si87Zr13, NiSi4, graphitized_carbon, C851Mo1700, nanofilamentous, Pd24Pt, carbide, CsHNOP, C49Zr50, CuH2Ti, Cfe3Mn3, titanium_carbide, Al555Co93Si352	Graphite, nitrogen, boron, nickel, iron, oxygen, silicon, C, organic, cobalt, metal, copper, titanium, hydrogen, Ni, content, CSi, surface, Fe
${E}_{carbon}^{glove}$	Dioxide, emissions, CO2, greenhouse, gases, sulfur, emission, monoxide	Gas, global, energy, increase, cut, less, water, tax, change

**Fig. 4:**
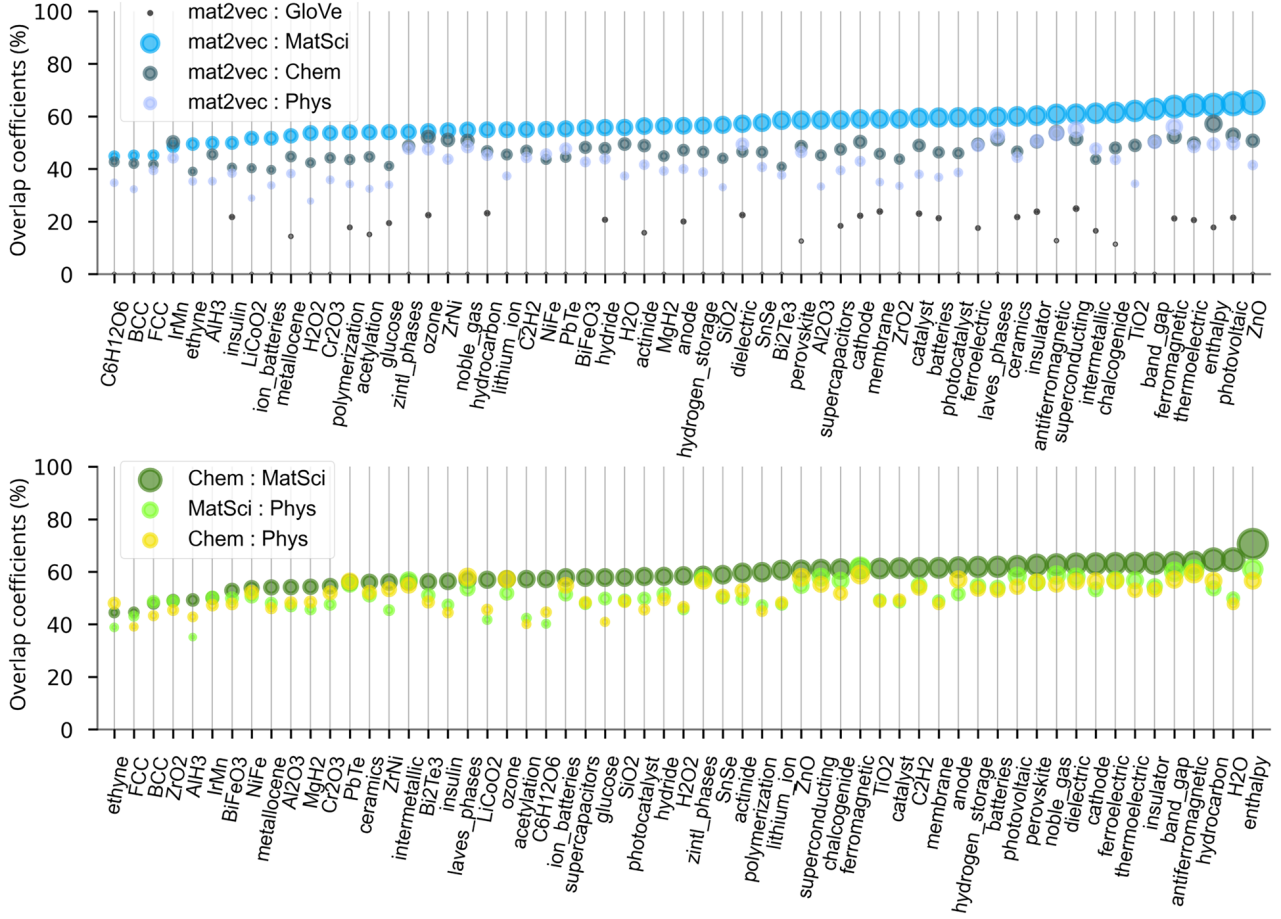
Comparison of the Szymkiewicz–Simpson overlap coefficients of embedding subspaces, originating from different domains, generated using 1000 vocabulary tokens and the first 500 tokens, for each high-frequency prompt, as indicated on the x-axis

This preliminary qualitative assessment of narrowly constrained embedding subspaces, such as those in the provided example, provided us with tentative insights into the structural composition of vocabularies and the overall variability of associated word embeddings. Accordingly, to systematically quantify these observations, in Fig. 4 the overlap coefficients, calculated when the size of the vocabularies and of the embedding subspaces were randomly limited to 1000 and 500 tokens, respectively, were presented.

The visual representation includes proportionally sized data points to reflect the calculated intersection percentage and emphasize the degree of overlap. The upper graph illustrates the intersecting patterns between all embedding subspaces per prompt, while the lower graph focuses exclusively on the intersections between custom embedding subspaces. As evident from the figure, the predominant majority of coefficients exceeds 50% in both graphs, with the most extensive overlap manifested between Mat2Vec and MatSci in the upper graph and between MatSci and Chem embedding subspaces in the lower one. In addition to the MatSci subspace, the Mat2Vec-based subspace also demonstrated a substantial similarity to the corresponding chemistry-related and physics-related subspaces, with coefficients exceeding 35% and 40%. On the other hand, the intersection between the two baseline vocabularies was found to be less than 30%, a finding consistent with the expectations, given the technical sophistication of the training corpora and fundamentally different algorithmic methodologies by means of which models were developed. Apart from several exceptions, the majority of custom embedding subspaces exhibited mutual overlap exceeding 50%. The mean overlap coefficients calculated for the intersection of baseline vocabularies with custom vocabularies averaged across all prompts equaled 38.50%, while that calculated for the intersections only among custom subspaces equaled 53.41%. This substantial overlap was initially attributed to the content similarity of the source documents and the application of identical protocols for pre-processing training manuscripts. However, at this point, it is critical to emphasize that this analysis incorporated only 500 tokens from embeddings subspaces, drawn from a pool of 1000 vocabulary tokens. When vocabulary constraints were removed, allowing unrestricted token participation in the generative processes of embedding subspaces, the averaged intersection coefficients decreased dramatically to 7.84% for the upper graph and 11.69% for the lower one.

**Fig. 5:**
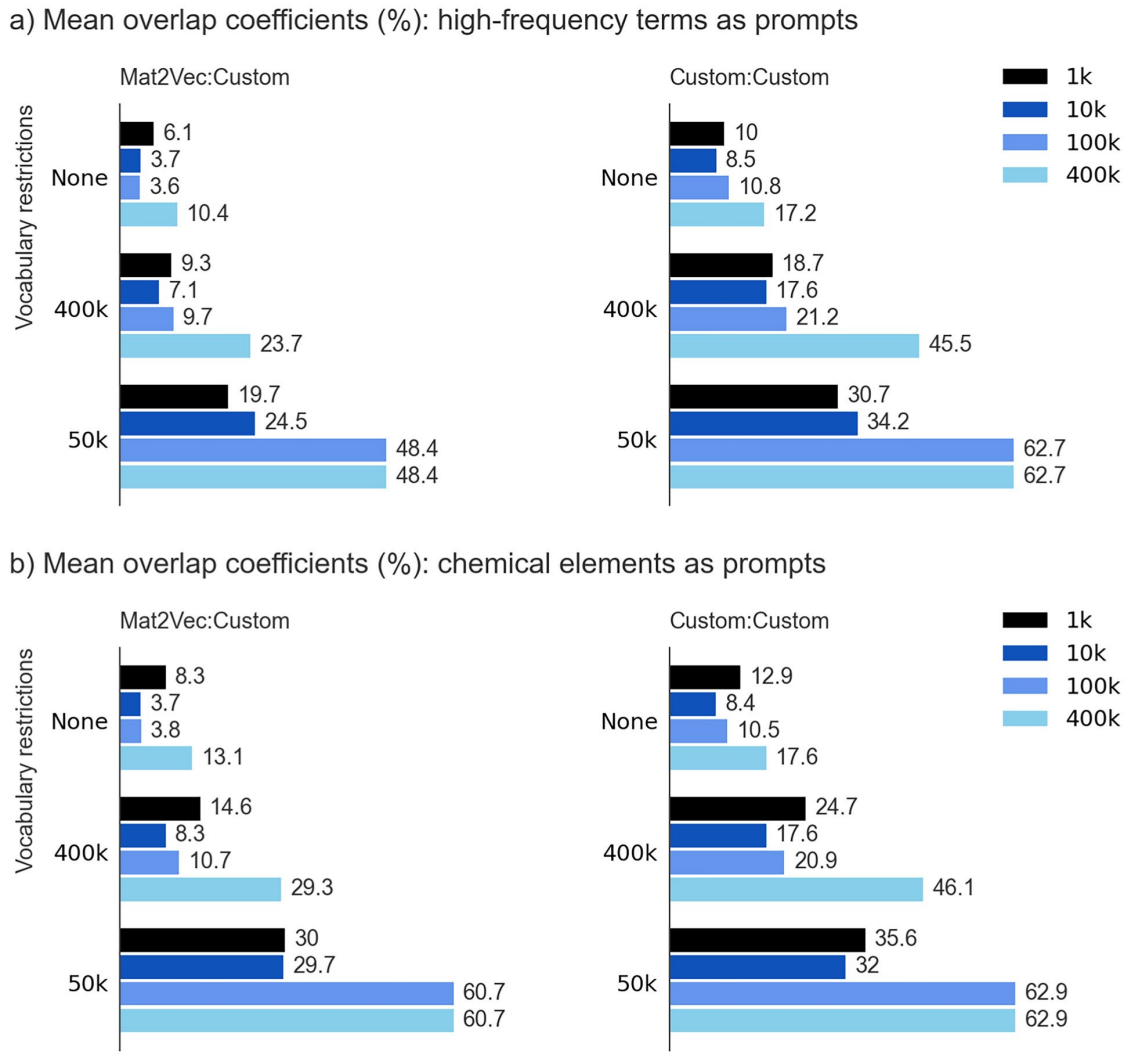
Mean overlap coefficients per prompt given four sizes of embedding subspaces (1k, 10k, 100k, and 400k) and three types of vocabularies (50k, 400k, and unr): **a** Coefficients are calculated using high-frequency terms as prompts; **b** Coefficients are calculated using chemical elements as prompts

This dramatic decrease in average overlap coefficients observed across all restrictions imposed upon the variability and embedding subspaces is presented in Fig. 5(a). The mean overlap coefficients reflecting the intersection of Mat2Vec baseline with custom vocabularies, alongside those reflecting the intersection among the custom sets of vocabularies, span twelve previously defined configurations of subspace embeddings, each generated using high-frequency prompts. In parallel, Fig. 5(b) exhibits analogous coefficients, derived under identical parametric constraints, using the first 95 chemical elements from the periodic table rather than high-frequency prompts as generators of subspace embeddings.

Comparative analysis of all figures reveals a notably larger degree of overlap between custom vocabularies, which is a phenomenon that could be attributed to several methodological factors: the systematic collection procedure of the training documents, their comprehensive format, and the implementation of the standardized methodology used for pre-processing of the assembled corpora. Accordingly, while materials science, physics, and chemistry-specific corpora indeed exhibit divergent ontological characteristics, the unified formatting procedure and tokenization apparently facilitated a more robust association between prompts and their respective semantic neighborhoods. The mean intersection coefficient exhibited a pronounced dependency on both the extent of embedding subspaces and on the restrictions imposed upon the vocabulary size – constraints that inherently reflect the lexical composition of each embedding subspace. When the top 50,000 tokens from vocabularies were used in the generating procedure of the embedding subspaces, each observed intersecting set contained far more shared tokens than when expanded to 400,000 or beyond. This observation is not surprising, as it unquestionably originates from the fact that the majority of tokens when ranked by occurrence frequency, tend to signify general terms that routinely appear to frame the discourse rather than carry the specialized content. Nevertheless, one should be careful not to overinterpret this finding. The significant presence of the general, common content terms does not imply an absence of specialized terminology or words with explicit materials science functionality within the 50 k-embedding subspaces; rather, it demonstrates that most of the tokens were those found in any document across the range of studied disciplines.

Upon relaxing the restrictions imposed upon vocabularies to incorporate the first 400,000 tokens into the respective embedding subspaces, the average overlap coefficient markedly decreased, as evident from Fig. 5. Tokens found in such expanded vocabularies, arranged according to occurrence frequency, represented a remarkable confluence of terms universally occurring in any document, domain-specific or otherwise, and of those highly specialized for studied domains, such as chemical and mathematical formulae, measurement units, and physicochemical entities that fundamentally affect the nature of embedding subspaces which they populated. The complete removal of restrictions, effectively permitting any vocabulary token an equal probability of being considered as neighboring to a given prompt, led to the further minimization of the intersection of embedding subspaces. This phenomenon implied the increasing orthogonality of embedding subspaces despite them being trained using conceptually related and similar corpora.

A critical aspect of this framework is recognizing that chemical formulae exhibit substantial heterogeneity, ranging from those easily identifiable in any general knowledge documents to those with highly domain-specific characteristics in terms of the domain to which they relate and of the lexical shape representing their chemical composition. Consequently, an analytical approach that focuses on tokens exhibiting high or moderate similarity risks constraining the embedding subspaces to the point of systematic elimination of formulae that, while infrequently studied and rarely encountered in the scientific literature, may reflect a large degree of innovativeness related to materials design. These rare formulae could conceivably convey a core for potentially new scientific knowledge in chemistry and materials science. However, because they do not necessarily demonstrate pronounced similarity to a given reference prompt, they face potential exclusion from conventional analytical frameworks.

### Conservation and transfer of information

**Fig. 6:**
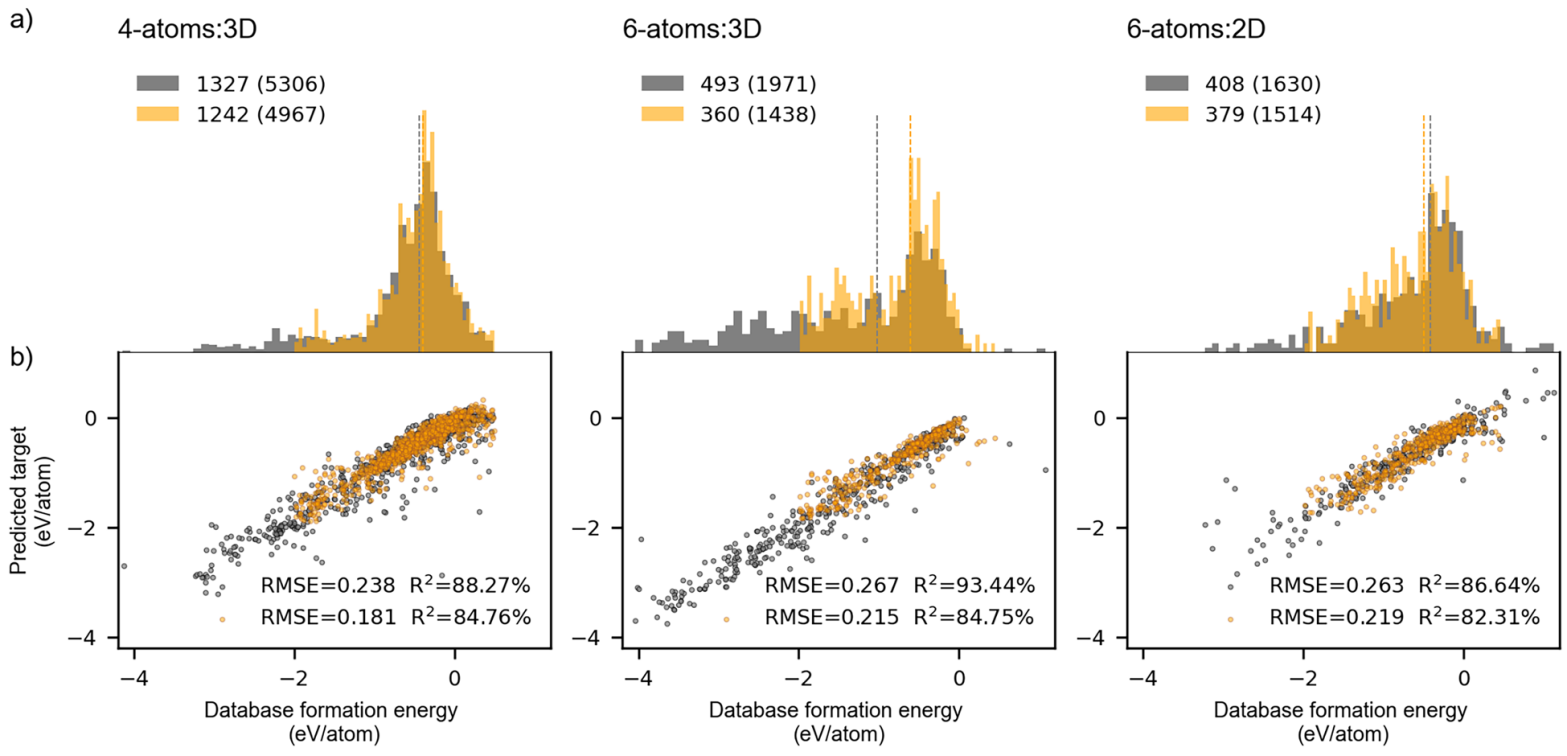
Independent test sets generated using MatSci200-type composite features: **a** Distribution density of target variable with indicated number of test (training) examples for (−4 eV/atom, 1 eV/atom) energy range (gray) and (−2 eV/atom, 0.5 eV/atom) energy range (orange); **b** Formation energy predictions plotted against database values

The effects of potential discrepancies among a collection of compounds gathered from different databases were controlled by selecting the same database from which samples of different sizes were drawn. A preliminary data set drawn from the Materials Project database consisting of 4 types of unique atoms per unit cell (4-atoms:3D) originally contained 7097 training samples (7097, 800). A data set consisting of 6 unique types of atoms per unit cell (6-atoms:3D) was drawn from the same database to assess the effects of different numbers of features, originating from different numbers of atoms, on the training process and subsequent generalization. The later data set, however, contained only 1971 training samples (1971, 1200), leading us to assume inferior preliminary generalization. Instead, both training and test errors of the 6-atoms:3D data set were significantly better than those of the 4-atoms:3D; on average, RMSE equaled 0.26 eV/atom and 0.81 eV/atom for 6-atoms:3D and 4-atoms:3D data set, respectively. Disregarding for the moment the extent of errors, which were large relative to the average ground truth values, we attributed this significant difference to the initial distribution of the target variable in the 4-atoms:3D data set containing formation energies ranging from -4 eV/atom to 4 eV/atom, unlike in the 6-atoms:3D, which was confined by the upper boundary of about 1 eV/atom. The unbalanced 4-atoms:3D data set contained an extensive tail diverging towards the positive energies of unstable compounds, with a notable number of outliers present among the negative values. Accordingly, a substantial number of examples found both within stable and unstable compounds lacked the necessary information to foster the learning process and were irrelevant from the viewpoint of the regression algorithm. Thus, the majority of unstable compounds were removed from the data set to match the energy range (-4 eV/atom, 1 eV/atom) of target variable from other data sets. This initial cut-off procedure made all prediction errors comparable, regardless of the types of embeddings used for materials’ representation. At this point, we further followed the recommendation by Blum and Langley[47] that the rate of learning is increased by focusing the model’s attention on informative examples and given that most examples were concentrated within the range of -2 eV/atom to 0.5 eV/atom, we reduced all data sets to that span. Following these sample manipulations, a distribution density of target formation energies became such that a similar skewed distribution could approximate all of them, as indicated in Fig. 6(a), where independent test sample distributions are provided before and after energy reduction from (-4 eV/atom, 1 eV/atom) to (-2 eV/atom, 0.5 eV/atom) range. The reader should bear in mind that the original distribution of the 4-atoms:3D data set is not presented here, as it was deemed irrelevant for further analysis. To address the generalizability of a regression model to lower dimension materials, the model’s performance on the data set comprised of two-dimensional compounds having six unique atoms (6-atoms:2D) was also tested.

As illustrated in the figure, constraining the sampled energy range resulted in a reduction in the number of examples within each data set. The most pronounced reduction was experienced by the 6-atoms:3D data set, as it exhibited a more uniform distribution of examples below -1 eV/atom compared to the other two data sets. Despite this reduction, the target focus improved the predictive capacity of the regression model across all three samples, as evidenced by computed RMSE in Fig. 6(b). In this figure, predicted target variable values are plotted against original database values obtained by training a regression model using features generated with materials science word embeddings consisting of 200 dimensions (MatSci200). Both computed RMSE and R^2^ are provided for each data set. The smallest RMSE of 0.181 eV/atom was achieved by the model trained on the largest 4-atoms:3D data set confined to an energy range (-2 eV/atom, 0.5 eV/atom), while the highest coefficient of determination, R^2^, equaling 93.44% was obtained from the model trained with a notably smaller 6-atoms:3D data set. Both outcomes straightforwardly stemmed from the structure of respective data sets. The former benefited from a large concentration of examples in a narrower range that surpassed the effects of the outliers present below -2.5 eV/atom, whereas the latter reflected a thick distribution of examples across the entire energy range. A noteworthy observation also arises when comparing two 6-atoms data sets. Namely, the computed RMSE in both unrestricted and restricted energy ranges are identical to two decimal places (0.26 eV/atom). On the other hand, in comparison with 6-atoms:2D, the R^2^ generally exhibits higher values for both the unrestricted and restricted 6-atoms:3D data set, though it remains nearly comparable when observed in restricted cases.

In order to put these results into perspective, we have compared the formation energies predicted using composite embedding features with those obtained using physically meaningful, human-interpretable set of descriptors. These reference features were automatically derived using chemical formulas via the matminer featurization tool, following the methodology established by Ward et al.[48] Employing the optimally selected physicochemical feature set on a dataset constrained to energy range of -2 eV/atom to 0.5 eV/atom, the random forrest regression model achieved a RMSE of 0.172 eV/atom with an R^2^ of 85.71% for the 4-atoms:3D dataset. For the 6-atoms:3D dataset, the model yielded an RMSE of 0.199 eV/atom and R^2^ of 87.25%, while the 6-atoms:2D dataset produced an RMSE of 0.187 eV/atom and R^2^ of 87.71%. In comparison, these performance estimates represent an improvement over results obtained using composite features, suggesting that conventional physicochemical descriptors still maintain a predictive advantage under the current experimental framework. Given that matminer features are explicitly designed to encode the well-established physicochemical relationships, the outcome favouring the physicochemical descriptors is not surprising. Howewer, the level of uncertainty in misclassifying the, e.g., thermodynamically stable materials as unstable, which would directly affect the synthesis procedures, remains comparable.

**Fig. 7:**
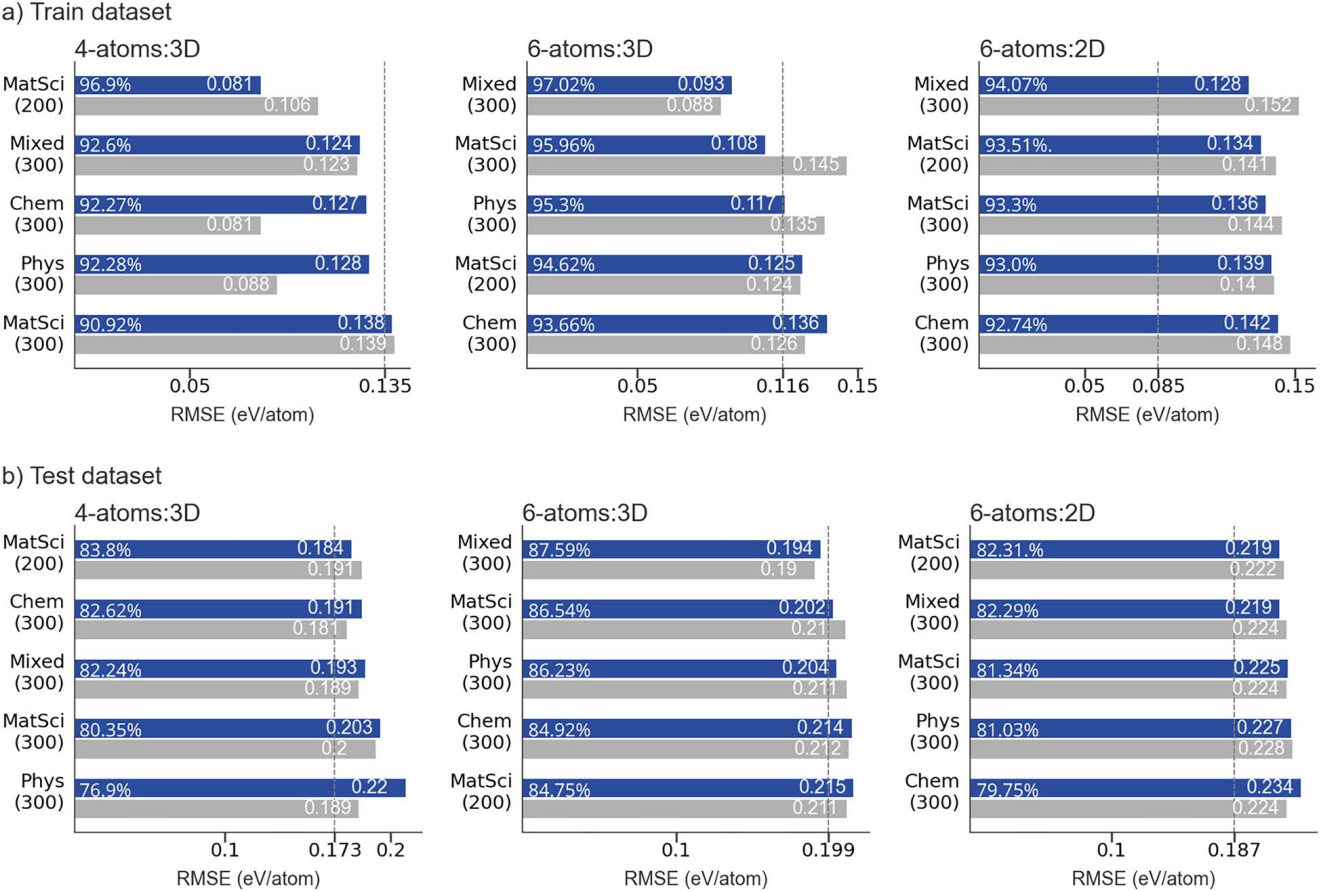
RMSE of 4-atoms:3D, 6-atoms:3D, and 6-atoms:2D samples, computed with original (upper blue bars) and reduced (lower grey bars) word embeddings and target variable constrained to energy range of −2 eV/atom to 0.5 eV/atom. Each horizontal bar emphases the computed RMSE value and R^2^. Vertical dashed line and corresponding value indicate a benchmark RMSE. **a** Training data sets; **b** Test data sets

A more comprehensive analysis of the model’s performance was conducted by incorporating the features generated using four additional types of word embeddings. The obtained RMSE and R^2^, computed using the training and the test data sets, is illustrated in Fig. 7(a) and Fig. 7(b), respectively, with the corresponding number of original word embedding dimensions indicated in parentheses. Figures also demonstrate the extent of errors obtained after compressing the feature vectors of composite embeddings to an optimal number determined by their univariate correlation with the formation energy, performed as described in the Methods section. In order to estimate the regression model efficacy in detecting the anomalies among compounds when featurized using composite embedding vectors, the reported computations were deliberately performed while incorporating materials identified as statistical outliers based on the percentage-error thresholds. This methodological approach revealed that identical chemical compounds were consistently categorized as outliers, independent of the origin domain of the word embeddings used to represent them, suggesting an intrinsic material-specific characteristic rather than a representational facet. Due to the inherent complexity of composite embedding representations of chemical compounds, along with the heterogeneous blending of multifarious materials into a unified dataset, a systematic investigation into the characteristics of outlier-classified materials requires further nuanced interpretation. This would be of great significance, given that outliers stemming from thermodynamic assessments could arise from both physical and methodological sources, e.g., experimental uncertainties in the datasets, the presence of different polymorphs or metastable phases, computational overestimations from first-principles, and more. Given composite embeddings' exceptional ability to detect outliers and indicate their presence in the dataset, regardless of the domain of word embeddings, outlier analysis could serve as a diagnostic tool for model performance. Thus, in the prospect of developing a predictive model with exceptional characteristics, using composite embeddings as material representations provides additional advantage. Another notable observation from the analysis of the predictions, was the potential of each domain representation to bias the regression model toward overestimating the number of thermodynamically stable compounds, i.e., those with negative formation energies. Nevertheless, a comprehensive characterization of compounds prone to being falsely predicted as stable and identification of generalizable, dataset-independent patterns necessitate investigation using expanded and more heterogeneous datasets.

Given the previously presented results obtained using MatSci200 features, the target variable in these computations was constrained to an energy range of -2 eV/atom to 0.5 eV/atom to ensure consistency. Accordingly, we will begin our analysis by scanning the Fig. 7 for RMSE obtained using MatSci200 features and comparing them with those obtained using MatSci300. An expansion of the feature set by an additional 100 vectorial components, originally imposed by hyperparameters tuning in the Word2vec model, had a variable effect on the magnitude of errors. In the largest 4-atoms:3D data set, one can observe such a significant training RMSE increase from 0.081 eV/atom to 0.138 eV/atom upon feature set enlargement that can hardly be attributed to the training stochasticity. Apart from MatSci300, other feature sets with 300 components – Chem300, Phys300, and Mixed300 – also produced notably larger training RMSE compared to that produced by MatSci200. This result suggests that embeddings with excess components within a given data set impair the training efficiency, which can be attributed to the likelihood that beyond a certain dimensionality threshold, additional vectorial components are strongly correlated and hinder the model refinement. Moreover, it is also evident from the plots that the model trained using 300-component feature vectors in this data set benefited from the dimension reduction during both the training and the testing procedure. The reduced Chem300 and Phys300 facilitated the substantial training RMSE reduction, while Mixed300 and MatSci300 benefited primarily from the considerable decrease in the compute time. Quantitative analysis revealed that based on their representations, 4-atoms:3D dataset, on average, contained 5.8% more compounds falsely estimated as thermodynamically stable. A particularly noteworthy point is the training error produced by the benchmark dataset, which amounts to 0.135 eV/atom RMSE, thus matching the lowest-performing MatSci300 training set. On the contrary, the generalization error produced by each custom data set followed the trend established by training performance, appart from the benchmark dataset, which proved to yield the best performing regression model upon an unseen data, as indicated in Fig. 7(b). Furthermore, the best RMSE for composite datasets was obtained in the case of MatSci200, and all feature sets with 300-component vectors advanced the regression model generalizability when reduced to an optimal number.

Compared with the 4-atoms:3D, we expected that a smaller 6-atoms:3D data set would produce greater training errors and inferior subsequent generalization due to fewer examples, regardless of the type of word embeddings. The results, however, varied and were even dependent on the dimensionality reduction process. Contrary to expectations, the training RMSE was substantially lower when a model was trained using the original Phys300, Mixed300, and MatSci300 data set. Conversely, Chem300 and MatSci200 produced more effective training on a larger data set. A generalization power of a model trained on a smaller 6-atoms:3D data set either outperformed the one trained on a larger 4-atoms:3D (see original Phys300 feature set in Fig. 7(b)) or within a margin of error yielded identical generalization (see both original and reduced Mixed300 and MatSci300 feature sets in set in Fig. 7(b). In the case of Phys300, however, the dimensionality reduction performed on the larger 4-atoms:3D data set benefited the learning process to the extent that the model managed to outperform the smaller one. The 6-atoms:3D dataset exhibited slightly improved performance in comparison to larger 4-atoms:3D dataset with a reduced rate of 3.1% for compounds being falsely estimated as positive in each representation. The observed generalization trends suggest that the design of these datasets does not guarantee improved predictive performance of a random forest model upon the inclusion of additional examples. The likely explanation is that a significant proportion of examples in selected data sets is concentrated within a very limited energy range. Accordingly, despite the size difference, many examples cease to advance the learning process of a random forest beyond a certain threshold, possibly even hindering it by introducing additional variability into the feature space.

A third 6-atoms:2D data set comprised of 2D materials yielded relatively inferior training and test RMSE and R^2^ values, yet comparable to those observed in the previously analyzed data sets. The magnitude of errors was also influenced by the type of word embeddings used in feature sets, and similar to the findings from the 6-atoms:3D, dimensionality reduction failed to impact the overall training process positively. Both 6-atoms:2D and 6-atoms:3D data sets were of comparative size, resulting in training sets of nearly identical structures, namely (1514, 1200) for the 6-atoms:2D materials and (1438, 1200) for the 6-atoms:3D data set. In contrast, the 4-atoms:3D data set of shape (4967, 800) contained over 3 times as many examples and two-thirds the number of vector components, making it appearently less comparable to the other two. From that perspective, one can justifiably challenge our previous analysis and comparability of used samples. The two-dimensional dataset yielded, on average, 7.2% more thermodynamically stable predictions than expected.

#### Data set compression via examples sampling

To offer a more appropriate comparison, we conducted additional experiments by randomly sampling 1440 training examples from the 4-atoms:3D data set and evaluated their impact on the regression model. In addition, we also performed a controlled sampling of the data points from the same data set to approximately match the distribution of the 6-atoms:3D sample. To do so, prior to splitting the data into training and independent sets, we balanced the number of examples present in an energy interval -1 eV/atom to -2 eV/atom for both 4-atoms:3D and 6-atoms:3D data sets and ensured that this number was approximately half the number of examples present in the upper range of -1 eV/atom to 0.5 eV/atom. While this approach arguably introduced an over-control of the data, it allowed us to investigate the influence of embeddings size on the regression model within a more controlled framework. Nevertheless, it is important to note that the sample size in this experimental setting is relatively limited, particularly for the independent test set. Additionally, the high dimensionality of feature vectors, relative to the sample size, introduces complexity that should be carefully considered when employing word embeddings as features within the limited data set.

**Fig. 8:**
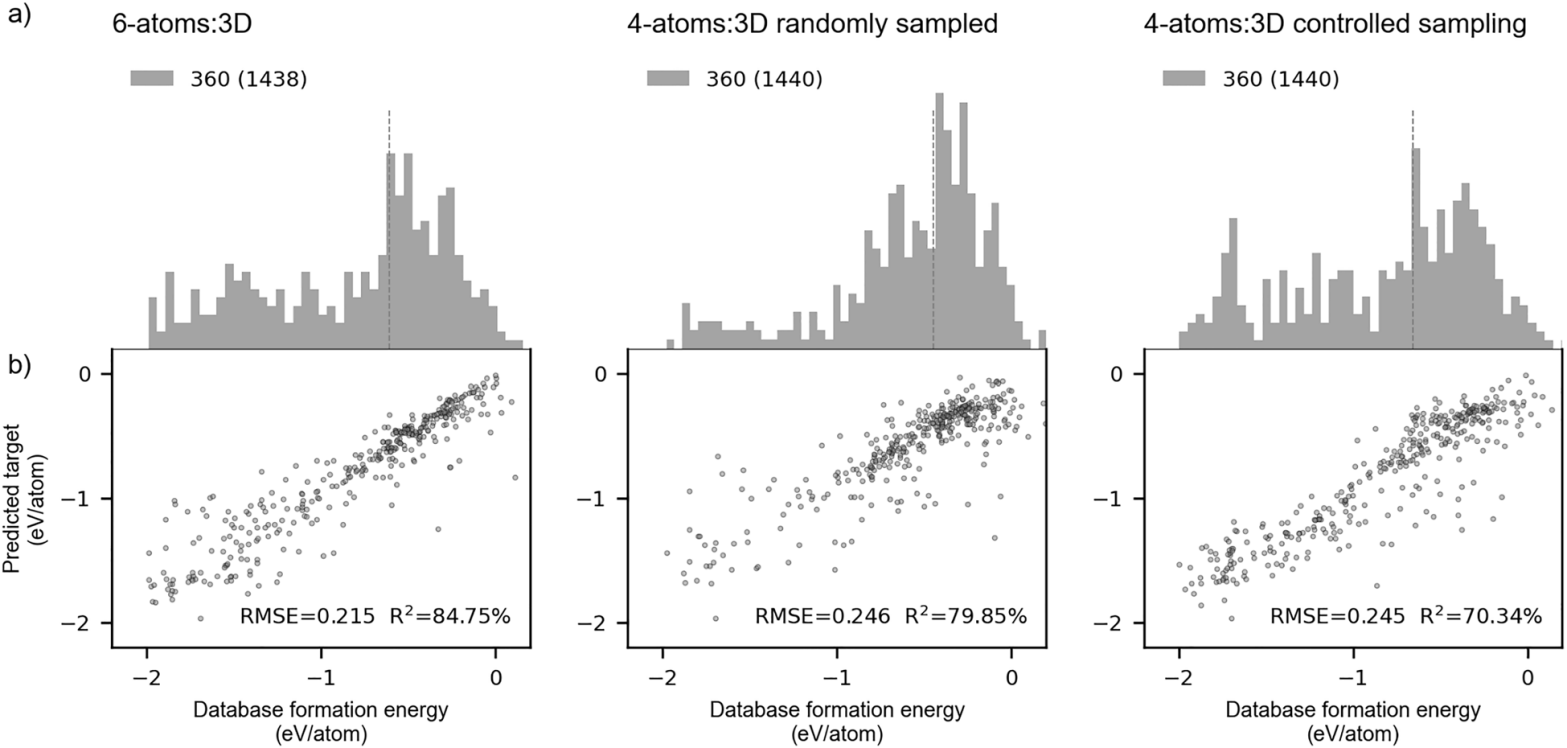
Independent test sets generated using MatSci200-type composite features: **a** Target variable distribution density from 6-atoms:3D and two sampled 4-atoms:3D data sets; **b** Formation energy predictions plotted against database values

The distribution densities of three independent test sets – the 6-atoms:3D, the randomly sampled 4-atoms:3D, and the 4-atoms:3D with controlled sampling – are presented in set in Fig. 8(a). Concurrently, set in Fig. 8(b) provides the model’s predictive performance quantified by RMSE and R^2^, reflecting its generalization capacity when trained on MatSci200 features. In contrast to a large-sized sample that increases variability and predictive potential by a myriad of introduced examples, a smaller-sized sample generally leads to pronounced variations in predictions. However, as evident from the graphical representations, apart from the reduced number of data points affecting the model’s training and generalization, a particularly striking effect on the model also arose from the number of unique atoms within the unit cell.

Before addressing this particular outcome, it is instructive first to compare the distribution of target variables derived from the 6-atoms:3D data set with those from the randomly sampled 4-atoms:3D and infer its potential influence on the regression model. In comparison to the 4-atoms:3D random sample, a target variable extracted from the 6-atoms:3D is somewhat more evenly spread across the studied energy range. Both data sets manifest a broad peak in the interval 0.5 eV/atom to -1 eV/atom, with a significant distributional divergence occurring below this range. As the RMSE and R^2^ derived from training the regression model on these two samples notably differ, especially R^2^, we presumed that this divergence originated from the corresponding divergence of the two distributions. Given that the majority of 1440 training examples from the 4-atoms:3D sample were predominantly drawn from an interval of -1 eV/atom to 0.5 eV/atom, the remaining -2 eV/atom to -1 eV/atom range remained underrepresented, exhibiting the insufficient coverage with training points. The sparsity of data points in one half of the studied target variable range, contrasted with their high concentration in the other, inevitably resulted in poor model performance and a high degree of dispersion in the predictions target variable. This outcome was clearly reflected in the obtained results; by comparing the RMSE and R^2^ values between the 4-atoms:3D and 6-atoms:3D samples, we see that the former data set produced strikingly inferior test errors. Seeking to mitigate this, we revisited the 4-atoms:3D data set, applying a controlled sampling strategy as previously described. When sampling was controlled by weighing examples according to their concentration, the regression model demonstrated somewhat improved performance, albeit only in terms of the coefficient of determination. As the R^2^ signifies the distribution of the predicted target variable with respect to its ground truth values, this improvement was, expectedly, reflected upon the sample redistribution. Yet, even with this adjustment, its magnitude remained lower than that derived from the 6-atoms:3D sample. Moreover, the RMSE from 4-atoms:3D data sets remained invariant to the manipulation of the target distribution. The implications arising from these outcomes suggest that a data set filled with the configuration of compounds comprised of six unique atoms, yielding 1200 dimensions of corresponding composite embeddings is superior to the data set comprised of compounds with four unique atoms, yielding composite embeddings with 800 dimensions.

The effectiveness of a more extensive feature set originating from diverse atoms belonging to one compound instead of additional vector components per atom itself is evident from the present computations. A greater number of atoms per compound increases the activation potential of embedding components relative to the target variable via a large distributed feature set in a data set with enough examples, while reducing the interference from coupling or interdependencies among components from individual atoms. The model, thus supported by a broader array of active components to choose from, more effectively learns to recognize the patterns within the data, ultimately leading to more robust and generalizable predictions. This conclusion implies that a data set populated with word embeddings of atoms composing large compounds is sufficient to generate an eligible training set for a random forest regression model. However, the question of how large individual compounds ought to be for the optimal training setup remains here unanswered, partially because it must be addressed via experiments encompassing different machine learning architectures, such as deep neural networks, for straightforward generalizable conclusions to be drawn.

#### Data set enlargement via 2D and 3D examples merging

With that said, the reader should keep in mind that the sample size, especially relative to the feature set size, is relatively limited and that the 4-atom:3D data set exhibits a significant improvement of results with additional data points. Therefore, we propose that further investigation is conducted when notably larger or more diverse data sets are eligible. For the time being, though, to explore the potential of data set enlargement with examples drawn from an entirely different database, the original 4-atoms:3D sample was augmented with a corresponding 2D sample composed of 4 unique atoms (4-atoms:2D) to match the structure of the original data set. Integration of 2D examples resulted in a total of 5025 training and 1257 test examples upon the train-test split procedure, with 615 and 154 2D examples populating the train set and the test set, respectively. In addition to studying the effects of such enlargement, this experimental setup contained another distinct investigative opportunity. Namely, the one addressing the prospect of random forest learning to differentiate between 2 and 3D compounds, as they manifest notably distinctive physico-chemical characteristics.

**Fig. 9:**
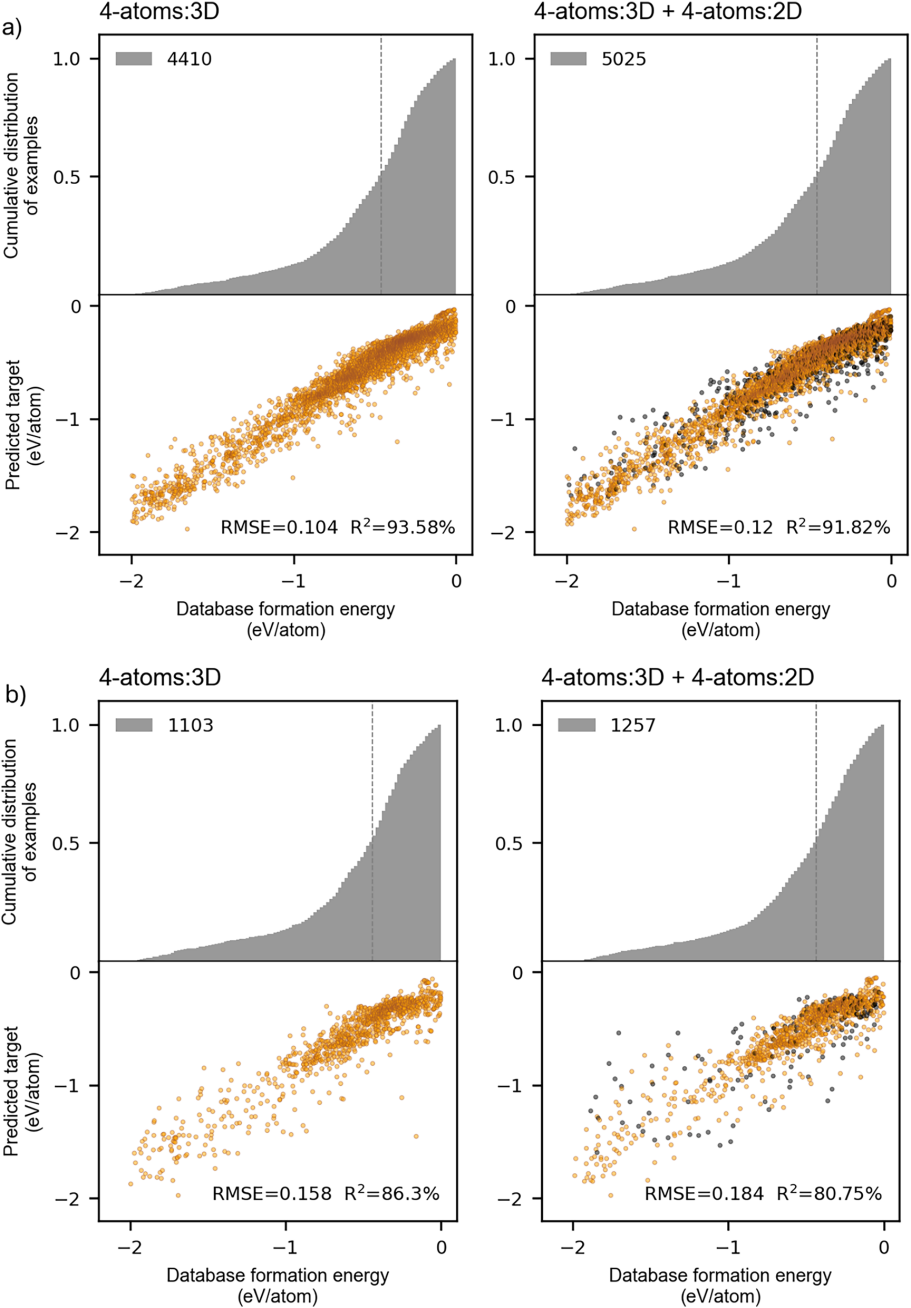
Cumulative distribution density of exmples relative to the distribution of target variable predictions obtained using MatSci200-type composite features representing the original 4-atom:3D and the augmented 4-atom:3D + 4-atom:2D data set: **a** Training data set; **b** Test data set

To focus optimization process of a regression model, we excluded the aspect of materials’ instability from the learning process by restricting the target variable to negative energies. The results obtained from the training and testing procedure using the original 4-atom:3D data set, as well as the augmented 4-atom:3D + 4-atom:2D data set, are presented in Fig. 9(a) and Fig. 9(b).

Instead of the distribution density we opted for the cumulative distribution of two samples. Comparison of these graphs reveals that the inclusion of 2D structures did not significantly modify the distribution of the target variable, neither in the train set nor in the test set of the original 3D sample. The majority of formation energies remained concentrated between -1 eV/atom to 0 eV/atom, with a large tail extending toward compounds with more pronounced stability (more negative formation energies). Accordingly, the possibility of data clustering that could severely disrupt the computations was excluded. The behaviour of the augmented sample was expected to match that of the original sample and the comparison of the two was, thereby, justified. Nevertheless, the newly added 2D stable examples approximated only 12% of both the unified training set and the unified test set. With this in mind, if the 2D examples, with their accompanying composite embeddings, were compatible with the 3D examples, we expected that the performance of the regression model would at least slightly improve due to additional data points. Conversely, if the two data sets contained incompatible examples, the model’s training capacity and generalizability could remain invariant to the augmentation due to a small number of added examples or, depending on the level of incompatibility, they could degrade.

As illustrated in the Fig. 9, a small degradation in the computed RMSE and R^2^ was observed during the training phase, with a more pronounced deterioration evident during the model’s generalization evaluation. The coefficient of determination computed using the test set mainly reflected the augmentation effect manifesting in a thick dispersion of predictions. The limited percentage of 2D examples relative to 3D examples in the unified training set proved insufficient to improve the model’s learning capabilites; rather, it caused a disturbance in the pattern recognition process. Furthermore, as the majority of data points deviating from the linear trend of perfect prediction are those associated with the 2D samples, it is reasonable to assume that the model detected some inherent underlying distinctions between feature sets of 3D and 2D composite embeddings. Even though we cannot state with great scientific certainty that the difference in results originated from the encoded chemical information, we can conclude, however, that this observation reflects the influence of data manipulation on the random forest regression model and, consequently, calls for further investigations when larger materials samples are readily available.

## Discussion

Since the alignment of NLP and LLMs with materials science and chemistry research, only a few language-based materials recommender systems have used stand-alone word embeddings as composite machine learning features in materials design. Most research still focuses on obtaining a broad spectrum of material analogs by measuring the cosine of the angle between vectorial representations of compiled materials with vectorial representations of materials having the desired characteristics. Such spatial similarity, however, points to the semantic similarity between materials' tokens, rather than straightforward similarity between their innate chemistry. Therefore, accessing the presumed chemical information rooted in materials' embeddings and recognizing that they share fingerprint details would allow for asserting chemical equivalency and labeling materials as uniquely interchangeable. However, decoding such specialized chemical information currently represents a technical challenge. Another limiting aspect is the substantial heterogeneity of chemical nomenclature, ranging from formulae commonly found in specialized and general knowledge literature to those exhibiting pronounced domain specificity. This assortment introduces a disproportionate information density from which a language model extracts data, thereby hindering the identification of materials with high or moderate vectorial proximity to the materials rarely researched or documented in the literature. Another attention-deserving aspect is that the angular-based alignment of materials' representations reflects their inherent variability. According to the definition proposed by Burdick et al. [35], the observed variability reflects inconsistencies among the sets of nearest neighbours of a token randomly extracted from vocabularies generated from corpora containing different training inputs. As shown in this work, the variability of static embeddings is significantly influenced by the origin of the training documents of the parent domains. Whether this is a relevant and concerning facet of the already variable, context-dependent embeddings is beyond the scope of this research; nevertheless, given that it could potentially lead to fluctuations in the established similarity of materials across training corpora, it merits significant attention. Moreover, the nuances in the complexity of word-level representations of chemical compounds introduced by newer, e.g., transformer-based, language models are multifaceted, as described in the Methods section; thus, they require substantial supplementary analysis.

The rationale for employing static embeddings of atoms instead of finalized compounds, as autonomous machine learning features for predicting properties of newly designed materials, which they are a part of, lies in their inherent versatility. Throughout the manuscript, we have demonstrated their flexible nature and indiscriminate utility, given the accessibility and tractability of the encoded chemical details. As presented, the word embeddings of atoms could be employed in their native format, directly synthesized by the language model, or conversely, subjected to prior modifications that align them with the specific requirements of a given design task to enhance the precision of the model's prediction. For example, one such advantageous modification—not directly explored by this research—could involve augmenting the embeddings with numerical padding to complement a training set with information reflecting physical constraints or conditions under which the desired materials are required to be stable. This inherent plasticity enables their seamless integration with other data modalities, generating a symbiotic configuration that is especially useful if compression via dimension reduction is required. As results indicate, the dimensionality reduction of composite embeddings does not interfere with intrinsic informational integrity, provided the dataset is sufficiently large. This finding is exceptionally relevant in the context of modern large language models that would yield much larger and complex embeddings of atoms, if they are to serve as autonomous machine learning features.

The complexities introduced by numerous vectorial components concentrated in joined atomic embeddings could be addressed using multiple complementary dimension reduction procedures, each providing a training set with distinct attributes predisposed for concrete design challenges. It should be noted that the primary approach could still include calibrating the original hyperparameters of the language model to generate smaller-sized text embeddings. However, the embeddings may inadequately encode extensive information about the atomic properties or their behavioral patterns under specific environmental conditions. An alternative strategy could involve generating very large embeddings of chemical elements initially arranged in a data set reflective of the periodic table of elements, which would undergo selective reduction prior to their integration into compound representations. The non-specific filtering could then be used to eliminate low-variance predictors, for example. However, while computationally efficient, this approach only handles language model intricacies related to the periodic table of elements, potentially overlooking the properties arising from explicit mathematical or implicit chemical interactions. By prioritizing the individual atomic embeddings over their collective behavior within designed compound configurations, and irrespective of the target, the proposed practice might not prove optimal if atoms influence each other's representations when jointly processed by a learning model. Then again, the non-specific filtering could also be used to eliminate the low-variance vectorial components of atoms within compound arrangements; however, seeing that this is the artefact of the data set, it might introduce complications during the testing stage if newly composed materials are not fully reflective of the training set.

In contrast to both the information-restrictive and the atom-centric method, a third approach was effectively used in this research. It involves a posterior reduction of features subsequent to the arrangement of joined atomic embeddings into a training set, guided by their correlation with the chosen target. In general, this approach enables the compression of information-rich predictors to the smallest unit of the target-inspired representative vector; therefore, in addition to being the artefact of the language model, as well as of the composed training set, it incorporates the target-relevant information. Upon constriction the word embeddings can be augmented by adding design-related data, as previously indicated. In addition to augmentation padding being in the form of, e.g., a separately trained sentence embedding stating the design requirements, it could also present the conditions from published tabular data, images from experiments, and more. However, different modalities often exhibit divergent statistical properties, making their relationships non-trivial. Regardless, this is a significant advantage of the proposed methodology, which we did not address within this research; we simply presented it as an idea that could be explored in future work.

An important limitation of the proposed approach was the inherent shape of the data sets. More precisely, the stoichiometry of all compounds under investigation had to be identical throughout both the training and the test sets to avoid having unequal lengths of the feature set arrays. This complication simplified the feature set-generating process but severely limited the number of compounds available for training. In general, there could be numerous routes by which this drawback could be handled, preferably in the context of the research task that needs to be further optimized. In contrast, when this challenge is overcome, it should be recognized that the size of the independent test set, generated by joining atomic representations in an unrestricted myriad of different compositions, could be limited only by the requirements of materials under design.

## Conclusions

The investigation outputs an insight into the universal generalizability and potential applicability of domain-specialized word embeddings as high-dimensional machine learning predictors in computational chemistry and materials informatics. Building upon the previous studies, the research confirms the inherent variability that permeates static word embeddings of physicochemical entities and chemical elements due to information heterogeneity within the language model training corpora, when sourced from complementary and comparative scientific domains of materials science, chemistry, and physics. The observed persistence of the resulting variability highlights the exceptional sensitivity of the embedding process and the fact that the angular-based alignment of materials' language representations, based on which material analogs are usually drawn, should be addressed with exceptional care. The present investigation focuses on static embeddings as the primary representational framework due to their computational efficiency and established interpretability. However, this should not imply any advantage or priority over other, more advanced large language models, such as transformer-based architectures, which offer impressive capabilities in capturing complex linguistic relationships. Moreover, recent advances in the contextual language models, their increasing availability and usability demonstrated especially in the design of digital twins through angular alignment of their linguistic representations, underscore the need for extending variability analysis to these sophisticated architectures, and their benchmarking against static models across diverse materials science, chemistry, and physics applications. Accordingly, implementing the variability thresholds as rigorous quantitative metrics in automated pipelines and developing standardized benchmarks for acceptable levels across different application contexts is recommended.

We suggest applying domain-matched embeddings when predictive precision constitutes a critical requirement, particularly during the final stage of candidate selection for experimental validation, as domain-specific embeddings demonstrate acceptable performance. This recommendation gains particular significance given our observation that training corpus domain selection directly affects the thermodynamic stability prediction and accuracy. Moreover, the acceptable error in the predicted energies depends strongly on the application for which the materials are suited, since different phenomena operate within their specific energy scales and sensitivities. Accordingly, for screening materials that require specialized screening applications, a carefully curated corpus assembled from relevant subdomain literature should, without exception, outperform the massive, general-purpose collection of documents. This targeted approach not only preserves semantic coherence within specialized technical domains but also yields computational advantages.

Presented findings demonstrate that employing word embeddings as independent machine learning predictors of materials stability yields reliable results despite being affected by a broad and diverse training environment. Arguably, however, the observed variations in predictive accuracy can be attributed to both the diversity of source training material, as well as to the limited size of the data set. Furthermore, it was established that word embeddings exhibit remarkable flexibility, which enables their functioning in the original form, native to the Word2vec model, and post-subjecting to transformations aligning them with specific design requirements—a characteristic that significantly enhances their utility across various materials informatics applications.

This work provides an introductory guidelines for deployment of language-derived representations in automated materials discovery, where embedding variability can directly translate into misdirected experimental validation efforts. Methods for controlling the variability and tolerating its consequences could be systematically developed by identifying its source, which would ultimately enhance the functionality of natural language-based representations of atoms, molecules, and compounds. With such advancements, word embeddings would evolve from abstract technical outputs to robust autonomous feature vectors, and the physicochemical complexity of chemical elements, encoded via statistical patterns of their representative word embeddings, would provide universal, indiscriminate, and adaptable predictors.

## Methods

### Documents collection, text processing, and overview of domain-specific vocabularies

To obtain an ample number of diverse, domain-specific scientific documents, The Semantic Scholar Open Research Corpus (S2ORC) database, an aggregate of 81.1M open-access academic manuscripts from verified digital archives primarily written and published in the English language, was used [49, 50]. We found the S2ORC helpful because it contains manuscripts’ metadata, allowing the selection of scientific documents with different backgrounds. Thus, the respective domains of selected documents were identified, using the metadata tag, corresponding to three research fields – chemistry, physics, and materials science – containing only full-body text for an addendum to the corpus. After removing duplicates, each document was partitioned into sentences that populated the corpus, a simple text file with one sentence per line. This first pre-processing step differed from the equivalent step in the work of Tshitoyan et al.[8], whose guidelines were used to compose a corpus for ML training. The reason is the support for the considerably lengthier full-text manuscripts rather than abstracts arranged as one abstract per line of the corpus file as used in the cited work. The document segmentation into individual sentences was performed using the SciSpaCy tokenizer[28], a package based on models from the widely used spaCy library adapted for processing biomedical, scientific, and clinical manuscripts. Despite its focus on the biomedical domain, we opted for the en_core_sci_sm tokenization model. Splitting raw text into sentences was followed by splitting each sentence into individual tokens using the ChemDataExtractor tokenizer [51], with all tokens lower-cased except chemical formulas (H_2_O, MgH_2_) and units of measurement (^0^C, kJ/mol, A/m^2^). Redundancies such as commas, periods, brackets, and duplicates were removed. Tokens with a minimum count threshold of 5 were removed from the corpus to facilitate the focus on words with a moderate and high frequency of occurrence. This step was critical due to the participation of exceptionally rare chemical formulae, whose occurrence could introduce complexities during tokenization and interfere with the training procedure; moreover, this threshold enabled the removal of any potential and existing spelling errors. The size of the corpora was dictated by an observation that a sufficiently small and overly distinctive, idiosyncratic corpus worsens the quality of generated word embeddings. All corpora contained roughly 3.6 million tokens, with approximately 900k unique terms. Such a consolidated sequence of distinctive tokens – vocabulary – characterized by reduced noise facilitated the advanced rooting of actionable knowledge across representative vectors. In addition to the three mentioned corpora, a much larger corpus (mixed) of about 7.7 million tokens was generated as a mixture of documents in the following percentage: chemistry:matsci:physics = 23:32:45. The number of tokens in this corpus was about 53.22% larger than the average number in the other corpora. In comparison, the vocabulary size elicited from mixed corpus was about 46.67% larger than the average vocabulary size obtained using any other. For additional analysis of the effect a smaller-sized word embeddings have on their utilization as feature vectors in ML-based materials design, primarily referred to in the second part of the Results section, a materials science corpus containing approximately 1.7 million words was also generated.

The number of used documents, generated sentences and tokens per corpus, as well as the vocabulary features, such as the number of unique tokens and the vocabulary density (percentage of tokens remaining post-pruning given relative to initially obtained tokens), are provided in Table 3. The heterogeneous balance of corpora was maintained by providing an adequate mix of diverse scholarly articles, with the absolute number of articles from the given domain assumed insignificant as long as the total number of generated tokens aligned across corpora.

**Table 3:** Specifications of custom corpora and corresponding vocabularies

Research disciplines	Mixed	Chemistry	Physics	Materials science	Materials science
Word embeddings dimensions	300	300	300	300	200
Number of documents	700k	160k	300k	300k	100k
Number of sentences	88.9M	19.7M	45.9M	36.7M	11.9
Corpus size	7.7M	3.6M	3.6M	3.6M	1.7M
Vocabulary size	1.8M	900k	900k	900k	540k
Vocabulary density (%)	23.35	27.19	25.21	27.46	31.15
Abreviation	Mixed300	Chem300	Phys300	MatSci300	MatSci200

### Word2vec model setup

The word embeddings were generated by means of the Word2vec[30, 31], a natural language processing technique comprised of language model architectures used for fast and efficient learning of distributed representations of words – word embeddings – from data sets containing billions of words. We employed the continuous Skip-gram model architecture, as implemented in the Gensim library[52], which maximizes the classification probability of the context words given the center word in a unique sequence of words. We opted for a negative sampling strategy within the Skip-gram, which differentiates the center word from the sample of noise words that included 15 negative samples for each data sample. This approach provided results directly comparable to those later obtained using the continuous Bag-of-Words architecture; however, it proved to be significantly faster to train.

The number of predicted context words surrounding the target center word, the so-called window size, was a tuneable hyperparameter tested for three distinct values: 5, 8, and 15. Furthermore, the relative occurrence and the potential usefulness of exceptionally rare words, which are usually discarded as they fail to provide valuable training information, had to be considered in the context of the current experimental framework. According to our estimation, a minimum frequency count of 5 could be considered an optimal value in line with the degree of chemistry and materials science specialization. The model’s learning rate, a step size at each iteration, was reduced during the training process from the initial value of 0.01 to 0.0001 in 30 epochs. Another equally important hyperparameter was the dimensionality of the vectorized representations of words, I.e., the length of word embeddings. To ensure consistency with the Mat2Vec baseline model, we tested the effect of 200 dimensions on the downstream tasks of embeddings trained using materials science-affiliated corpora. In addition, however, the effect of 300 dimensions was also assessed using all other considered scientific domains.

#### Model evaluation and accuracy

Following the procedure outlined in the work of Tshitoyan et al. [8], the algorithm’s accuracy was tested by tuning the hyperparameters to analogies from 15,000 grammatical and 15,000 materials science quadruplets. Accuracy was defined as the percentage of correctly determined analogies from the two unified materials science and grammatical sets (total accuracy) and from the single materials science set (partial accuracy). These lists of analogy quadruplets were adopted from the mentioned reference, both containing suitably split inputs for testing word embeddings originating from materials science and chemistry disciplines. The materials science quadruplets, however, introduced a drawback in that most of them only partially addressed terms from chemistry or physics. Thus, to integrate further refinements to the model’s performance, a set of about 700 additional chemistry-related phrases was also included.

Nevertheless, not all words and phrases listed as analogy quadruplets were expected to be contained within the generated vocabularies. Those missing from the vocabularies, referred to as out-of-vocabulary words (OOV) [52], were found to be in direct correlation with the selection of the vocabulary, as well as its size, making the final accuracy of the model directly contingent on the considered domain. Surprisingly, the lack of any correlation of OOVs was observed across domains, and regardless of the fact that the majority of quadruplets were designed to fit the materials science, the highest OOV percentage was derived from that very domain (15.4% for MatSci200 and 13.5% for MatSci300). In comparison, significantly fewer OOVs were found to be missing from Phys300 (7.8%), Mixed300 (4.7%), and Chem300 (3.5%).

**Fig. 10:**
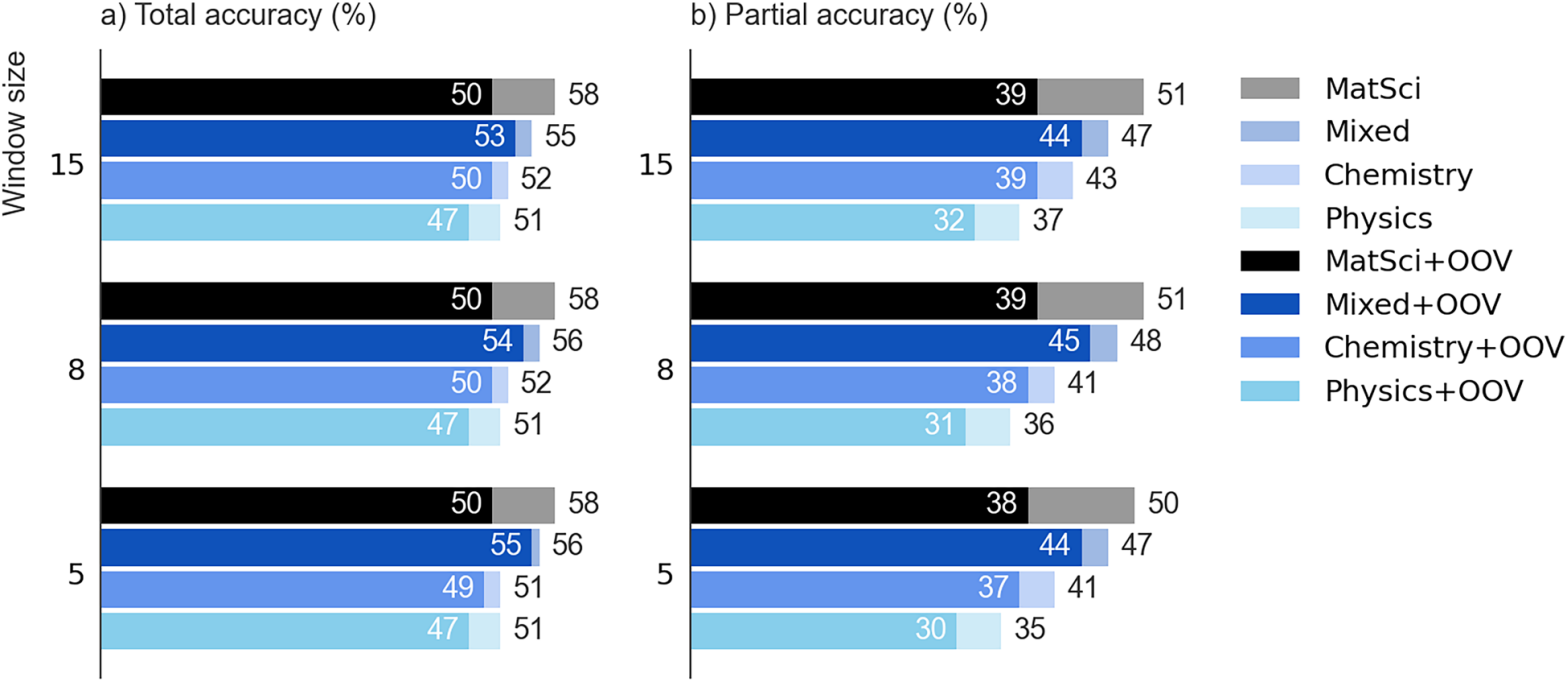
Word2vec model accuracy with and without OOV words included: **a** Total model accuracy; **b** Partial model accuracy calculated using domain-specific accuracy quadruplets

Figure 10 presents the computed total accuracy, as well as the partial accuracy of the trained model, with and without out-of-vocabulary words included. According to the results, the materials science corpus elicited the best-performing model in the case when OOVs were discarded from the assessment, which was deemed representative of our framework. On the other hand, when OOVs were included, the mixed corpus yielded the highest percentages, while materials science and chemistry corpora produced models with similar accuracies. Both total and partial accuracy followed the observed trend. The computed accuracy remained invariant to the different window sizes across all vocabularies, indicating its minimal effect on the training procedure. This outcome was exceptionally relevant, as it directly guided the selection process of vocabularies for the assessment of overlap effects and the subsequent variability of word embeddings.

#### Static versus context-dependent models

It should be emphasized that we opted for the Word2vec model over other available language models (e.g., MatBERT, MatSciBERT, et cetera.) given that analyzing bi-directional or uni-directional, context-dependent, transformers-based token embeddings requires particular attentiveness to the morphological structure of chemical terminology, as well as the context within which the representation of the selected word is trained, prior to extracting its representation due to several reasons. Instead of optimizing the representation of an integral word, in the training process of language models, embeddings of subword tokens are more commonly trained, which means that instead of getting an output per word, one obtains outputs for all of its subword components, which are then pooled into a unified representation. A typical chemical nomenclature, however, is brimming with exceptionally long multi-word chemical formulae layered with numbers, chemicals named after researchers responsible for their discovery or even some resembling particular English words (for example, 3,4,4,5-tetramethylcyclohexa-2,5-dienone, C60 – Buckminsterfullerene – buckyball, Josiphos ligands, Diethyl azodicarboxylate – DEAD). This intricate terminology complicates the tokenization procedure of materials and, even more so, the extraction of a learned representation of tokens and their subsequent unification. The reconstitution of subword token-level embeddings into a unified, coherent representation of a chemical compound requires an implementation of different consolidation strategies, each of which distinctly affects the downstream task performance [53]. The most commonly used mean pooling strategy, the arithmetic averaging of constituent subword embeddings, preserves underlying subword-level information while preventing the dominance of singular tokens. An alternative information-preserving strategy involves concatenating subword embeddings into a variable-length representation contingent on the number of subwords per word. Max pooling strategy focuses on extracting the maximum value across dimensions of subword vector embeddings, capturing the most variable features of each subword. The attention-weighted pooling approach employs a machine learning model to learn subword embedding weights based on their importance to the overall word meaning, then combines these weighted components into an integrated word-level representation. In first- or last-token pooling, only the embedding of the first or last subword token is used to represent the entire word, thereby discarding potentially crucial information through selective retention. Furthermore, the contextual representations of chemical nomenclature are inherently dynamic, as they adapt to the surrounding linguistic context. A single token, for instance "hydrogen", is represented with distinct embeddings in contexts "Materials for hydrogen storage" and "Hydrogen-based alloys for energy storage", potentially capturing nuanced physicochemical relationships. This contextual sensitivity, however, introduces both interpretative complexity and substantial computational overhead, as each new inference requires additional training. Further complexity emerges from the selection of transformer layers from which subword-level embeddings can be extracted, as they encode heterogeneous linguistic information depending on their position in the language model architecture, without exhibiting a monotonic increase in task-specificity across layers [54–57]. Lower layers, proximal to input, capture local syntactic patterns, word order, and basic grammatical structures. The intermediate layers encode the most transferable syntactic features suited for downstream tasks, while the higher layers are optimized for the semantic features of masked language modeling or next-token prediction. While the effect of layer-level complexities could, theoretically, be mitigated, for example, through weighted multi-layer combinations, the nuanced contributions of individual layers necessitate comprehensive empirical analysis to quantify their effects on word-level embedding variability.

These multiple complexities, encompassing layer-specific linguistic encoding, intricate contextual effects, and word differentiation into subwords using different tokenization schemes, in addition to large language models’ susceptibility to hallucinations [58], can collectively produce significant variability in word-level embedding representations of chemical compounds beyond domain-specific corpus selection. However, in the context where one could genuinely benefit from the use of contextual embeddings, for example, when context-dependent properties matter, such as reaction conditions affecting the physicochemical properties, these language models could offer significant advantages over their invariant counterparts. Having this in mind, to assess whether variability is a significant issue in these more recent types of language models, it is imperative to examine their individual and combined effects systematically. To that end, it should also be determined whether the established evaluation procedure for static embeddings can be directly transferred to (uni)bi-directional, context-dependent, transformer-based systems or whether it requires methodological adaptation for these frameworks. This analysis would require a substantial research endeavor; accordingly, we decided to focus this study on the benchmark effect of the static word embeddings, which maintain a fixed vectorial representation for each token, independent of contextual variations within a single training corpus.

#### Baselines models

To achieve the high-quality representation of chemical entities complete with exhaustive physicochemical information, the word embeddings were trained using full-text manuscripts, as they are generally broad in scope and incorporate variable domain-specific knowledge, thus providing a diverse information base for vectors’ training. This approach stands in contrast to the prior study, where trained word embeddings were produced using only abstracts of scientific manuscripts. While abstracts offer concise summaries, they inherently lack the depth and contextual breadth of full-text documents, which could be critical for capturing the nuanced relationships inherent in materials science domains. Regardless, to establish a comparative framework, the Mat2Vec word embeddings were adopted as the first-level baseline for comparison to those in the current study. The Mat2Vec vocabulary was selected due to its availability, demonstrated quality, and established representativeness within the materials science community. As the second-level baseline that further contextualizes presented results, the GloVe word embeddings (glove.6B.200d)[40] were also introduced. These embeddings, trained on a broad, general corpus of information, provide a domain-agnostic point of comparison independent of documents’ type and size, processing technique, algorithmic method, and contextual domain effect.

#### The Szymkiewicz–Simpson overlap coefficient

The intersection of the two finite sets of random variables, indicative of their mutually shared information, can be quantified by means of the Szymkiewicz–Simpson overlap coefficient defined for general sets[59]. Formally, this intersection coefficient is computed as the measure of intersection of two finite sets, A and B, normalized by the size of a smaller one; accordingly, it quantifies the shared information between two distributions as overlapping fraction given the smaller set of variables.

$$overlap\left(A,B\right)=\frac{\left|A\cap B\right|}{min\left(\left|A\right|,\left|B\right|\right)}$$

The overlap coefficient is bounded within the interval [0, 1], where a value of zero is characteristic of finite disjunctive sets, representing the absence of shared information, while the value of one signifies that between two sets, one is entirely subsumed within the other, reflecting the highest coefficient of shared information. Unlike other similarity measures, such as the Jaccard similarity coefficient for classical convex sets[60] which is sensitive to lengths of both sets under consideration, this formulation allows for comparison of distributions characterized by different lengths as it only relies on the dimension of the smaller one. Given the experimental framework described in this manuscript, where the generated vocabularies exhibited variability in size, this measure was found highly suitable.

To ensure consistency with the baseline, Mat2Vec, which used a context window size of 8, the overlap coefficient was calculated for token sets corresponding to word embeddings trained under identical conditions. This alignment minimized potential differences in contextual scope arising from the size of the learning context and focused the analysis on semantic relationships in the narrow domain-specific context relevant to materials science.

### Conservation and transfer of information

#### Databases

In the context of in-silico materials design facilitated by language processing and language models, the data sets of materials are usually assembled from a single publicly available database of materials and adequately split into a training/validation set for machine learning model’s optimization and an independent set for testing its generalizability. Depending on the database, the assembled data sets could contain materials with variable structural and electronic composition, those with diverse sets of attributes, performance, and functional traits. If such a data set is complete, a divergent machine learning model would learn a broad spectrum of highly variable information from which it could further generalize on an unseen test set. Conversely, the assembled data sets could also be comprised of materials of uniform applicability, structure, or composition, thereby enabling the machine learning model to learn a narrow set of very focused information. For the purpose of our research, we assembled several data sets comprised of various types of solid-state materials from two databases, one with exceptionally wide-ranging materials context and the other one with highly focused structural characteristics.

The first database from which a broad preliminary data set, consisting of four types of unique atoms per compound (4-atoms:3D), was drawn was the Materials Project database. More specifically, a subset of materials samples was drawn from the matbench_mp_e_form dataset, which is part of the Matbench v0.1 benchmark sets, adapted from the Materials Project database, for predicting DFT formation energy from composition. It was selected due to its extensiveness and completeness with electronic structure data, stability, and optical information obtained from basic electronic structure calculations [61, 62]. The effects of the database on the prevalence of information was controlled by selecting another set of materials from the same database, but with six types of unique atoms per compound (6-atoms:3D), thereby generating a complementary data set for comparison differing only in the input feature set size. 

In addition to the Materials Project database, a highly focused database containing two-dimensional compounds was used to assemble two additional sets of materials with configurations identical to those previously obtained (4-atoms:2D and 6-atoms:2D). Two-dimensional compounds are mostly a class of layered-structure materials, carbides and nitrides of transition metals with varying functional groups, recognised as a prototypes for atomistic design and chemical assembly from elemental building blocks [63]. Given various ratios of elements and distinct surface termination groups that can be combined, acceleration in investigation of these materials in silico offers huge potential for unlocking novel energy conversion solutions.

To generate an input representative of a chemical compound, a linear sequence of word embeddings of its constituent atoms, each consisting of either 300 or 200 vectorial dimensions, was composed. A composite embedding, as such, included a total sum of the number of embedding dimensions multiplied by the corresponding stoichiometric coefficient per atom. The resulting number of input features, generated using 300-embeddings or 200-embeddings, in the case of 4-atoms:3D data set equaled 1200 or 800, respectively, while in the case of 6-atoms:3D data set, it equaled 1800 or 1200.

The word embeddings of chemical elements were categorized into two types: embeddings of atomic symbols (${w}_{He}^{d}$) and embeddings of atomic names (${w}_{helium}^{d}$), both of which were trained and evaluated. The predictive potential of word embeddings for both representations was investigated, and it was established that embeddings of atomic names conveyed more nuanced information that provided slightly superior but comparable accuracy. As a result, only the results derived from atomic name embeddings were presented in this study.

#### Formation energy as machine learning target

We have opted for the formation energy of materials to represent a target value to be estimated using a random forest regression model. The formation energy is a physicochemical quantity indicative of a material’s thermodynamic stability, and it is a suitable ML target for evaluating the efficacy of machine learning models or the quality of assembled data sets due to its robustness to the majority of compounds. From a physicochemical viewpoint, formation energy represents a change associated with the formation of a chemical compound from its constituent elements under specified conditions. A positive formation energy signifies thermodynamic instability, whereas a more negative value correlates with greater stability. A distribution density of formation energy values is usually such that a similar skewed distribution can approximate most data sets, thereby simplifying the sampling process of an independent test set and facilitating the comparative analysis of results obtained from various databases.

When provided with a sufficiently large and well-balanced data set spanning an entire observed energy range, an ML model should be able to effectively learn the underlying distribution and accurately position new materials on an energy scale of thermodynamic stability. Moreover, it should be able to do that without explicitly indicating the physics of an energy release process solely from input features, which are, in the present case, abstruse high-dimensional vectors. It should be noted, however, that within selected databases, the formation energies were generated via mathematical models and first principles simulations. In some instances, these values may deflect from ground truth experimental values, depending on the utilized computational methodology or studied materials. Nevertheless, given that a flawlessly trained ML algorithm offering accuracy matching that of quantum chemical calculations or experiments was not the primary objective of this research, we have adhered to the assumption that the majority of existing deviations are sufficiently outnumbered and fused within the data sets, thus, do not interfere with the training process.

#### Random forest regression model setup

Composite compound embeddings representing various types of materials in the form of normalized features set were passed to a random forest regression model to learn to predict their respective formation energies. Given the relatively large number of features compared to the number of examples, particularly in the case of 6-atoms:3D and 6-atoms:2D data sets, roughly 20% of the total data set was set aside for independent testing of the regression model generalization performance.

Metrics used to evaluate the prediction accuracy and to control the possible overfit of this high variance model were the root mean square error (RMSE) and the coefficient of determination (R^2^). These measurements operate on different scales and provide complementary insights, therefore, the use of one should not necessarily exclude the use of another. The RMSE was strategically selected due to its sensitivity to expected outliers. This is generally considered a weakness of this measurement; however, as we wanted to be aware of the outliers’ presence in the data set, the RMSE was considered an optimal choice. Furthermore, the R^2^ value as a relative measure was exceptionally informative when describing a model’s predictive performance relative to the distribution of the ground truth values.

Randomized search five-fold cross-validation was used to estimate all hyperparameters and assess the model’s ability to generalize to an independent data set. Given the inherently stochastic nature of the random forest algorithm, a fixed random seed was required to ensure reproducible results, which was critical for maintaining consistency and reliability in the model evaluation process across different data sets.

#### Dimensionality reduction

The dimensionality reduction process served as an instrumental technique, alongside an ensemble of decision trees, to estimate the potential for computational efficiency enhancement and simultaneously moderate the curse of dimensionality – the overfitting caused by an excessive number of features. In general, the random forest performs well on correlated predictors in high-dimensional data sets; however, excess dimensions of joined embeddings of chemical elements introduce specific complexities via overly excessive noise and numerous inactive features or inhibit true predictors. Consequently, an auxiliary subset of features was generated by ranking predictors according to the level of correlation with the target variable [64]. A filter method selected for the reduction of features’ number was a univariate linear regression based on the Pearson correlation coefficient converted into an F-score, which individually computes the cross-correlation between each regressor and the target according to the formula:

$${r}_{x,y}=\frac{E\left[\left(X-{\mu }_{X}\right)\left(Y-{\mu }_{Y}\right)\right]}{{\sigma }_{X}{\sigma }_{Y}}$$

This exceptionally fast pre-processing step used characteristics of the training set for testing the effect each regressor had on a target, and given the large number of regressors that had to be sequentially tested, this technique proved advantageous over other possibly more precise.

## Data Availability

Generated corpora from all studied domains and trained models Chem300, Phys300, MatSci200, MatSci300, and Mixed300 are open-sourced and accessible at 10.6084/m9.figshare.28740341 and 10.6084/m9.figshare.28740122. The code generated for the purpose of the research is publicly available on GitHub at https://github.com/cat-of-schrdngr/informatter The containing README file in the GitHub repository provides information on the code and the project.

## Footnotes

1 The variability and the stability of word embeddings are considered mutually inverse attributes; in this manuscript, we will adhere to the use of the term variability.
